# Population-based input function for TSPO quantification and kinetic modeling with [^11^C]-DPA-713

**DOI:** 10.1186/s40658-021-00381-8

**Published:** 2021-04-29

**Authors:** Mercy I. Akerele, Sara A. Zein, Sneha Pandya, Anastasia Nikolopoulou, Susan A. Gauthier, Ashish Raj, Claire Henchcliffe, P. David Mozley, Nicolas A. Karakatsanis, Ajay Gupta, John Babich, Sadek A. Nehmeh

**Affiliations:** 1 Department of Radiology, Weill Cornell Medical College, New York, NY 10021 USA; 2 Department of Neurology, Weill Cornell Medical College, New York, NY 10021 USA; 3 Feil Family Brain and Mind Institute, Weill Cornell Medical College, New York, NY 10021 USA

**Keywords:** Population-based input function, Kinetic modeling, [^11^C]DPA-713, Normalization

## Abstract

**Introduction:**

Quantitative positron emission tomography (PET) studies of neurodegenerative diseases typically require the measurement of arterial input functions (AIF), an invasive and risky procedure. This study aims to assess the reproducibility of [^11^C]DPA-713 PET kinetic analysis using population-based input function (PBIF). The final goal is to possibly eliminate the need for AIF.

**Materials and methods:**

Eighteen subjects including six healthy volunteers (HV) and twelve Parkinson disease (PD) subjects from two [^11^C]-DPA-713 PET studies were included. Each subject underwent 90 min of dynamic PET imaging. Five healthy volunteers underwent a test-retest scan within the same day to assess the repeatability of the kinetic parameters. Kinetic modeling was carried out using the Logan total volume of distribution (*V*_T_) model. For each data set, kinetic analysis was performed using a patient-specific AIF (PSAIF, ground-truth standard) and then repeated using the PBIF. PBIF was generated using the leave-one-out method for each subject from the remaining 17 subjects and after normalizing the PSAIFs by 3 techniques: (a) Weight_subject_×Dose_Injected_, (b) area under AIF curve (AUC), and (c) Weight_subject_×AUC. The variability in the *V*_T_ measured with PSAIF, in the test-retest study, was determined for selected brain regions (white matter, cerebellum, thalamus, caudate, putamen, pallidum, brainstem, hippocampus, and amygdala) using the Bland-Altman analysis and for each of the 3 normalization techniques. Similarly, for all subjects, the variabilities due to the use of PBIF were assessed.

**Results:**

Bland-Altman analysis showed systematic bias between test and retest studies. The corresponding mean bias and 95% limits of agreement (LOA) for the studied brain regions were 30% and ± 70%.

Comparing PBIF- and PSAIF-based *V*_T_ estimate for all subjects and all brain regions, a significant difference between the results generated by the three normalization techniques existed for all brain structures except for the brainstem (*P*-value = 0.095). The mean % difference and 95% LOA is −10% and ±45% for Weight_subject_×Dose_Injected_; +8% and ±50% for AUC; and +2% and ± 38% for Weight_subject_×AUC. In all cases, normalizing by Weight_subject_×AUC yielded the smallest % bias and variability (% bias = ±2%; LOA = ±38% for all brain regions).

Estimating the reproducibility of PBIF-kinetics to PSAIF based on disease groups (HV/PD) and genotype (MAB/HAB), the average *V*_T_ values for all regions obtained from PBIF is insignificantly higher than PSAIF (%difference = 4.53%, *P*-value = 0.73 for HAB; and %difference = 0.73%, *P*-value = 0.96 for MAB). PBIF also tends to overestimate the difference between PD and HV for HAB (% difference = 32.33% versus 13.28%) and underestimate it in MAB (%difference = 6.84% versus 20.92%).

**Conclusions:**

PSAIF kinetic results are reproducible with PBIF, with variability in *V*_T_ within that obtained for the test-retest studies. Therefore, *V*_T_ assessed using PBIF-based kinetic modeling is clinically feasible and can be an alternative to PSAIF.

**Supplementary Information:**

The online version contains supplementary material available at 10.1186/s40658-021-00381-8.

## Introduction

18-kDa translocator protein (TSPO) receptor has been shown as a potential target for imaging neuroinflammation using PK-11195 PET [1–3]. Recently, a putative antagonist of TSPO, [^11^C]-N,N-diethyl-2[2-(4-methoxyphenyl)-5,7-dimethyl-pyrazolol[1,5-α]pyrimidin-3-yl]-acetamide ([^11^C]DPA-713), was developed concurrently with the TSPO agonist, fluoro-ethoxy derivative [^18^F]DPA-714 [4–6]. Both [^11^C]DPA-713 and [^18^F]DPA-714 were shown to have higher affinity than the first generation TSPO tracer [^11^C]-(R)-PK11195 [4, 7, 8]. Several studies have now demonstrated the usefulness of [^11^C]DPA-713 PET in quantifying neuroinflammation in different diseases, including multiple sclerosis (MS), Parkinson’s disease (PD), and Alzheimer disease (AD), both in animal and human studies [4, 9–12].

In PET, kinetic modeling is often essential for the accurate quantification of tracer uptake and metabolism in the tissue. This often requires the measurement of the tracer concentration in the arterial blood over time. However, this practice is often limited in terms of its invasive nature and associated risks to the subjects, as well as risky blood sample handling [13]. The need for inserting arterial lines in patients leads to significant discomfort and patient burden. In practical clinical trial settings, this often proves a key workflow bottleneck and can also adversely influence subject cooperation and accrual [14].

An alternative technique such as an image-derived input function (IDIF) [15, 16] or population-based input function (PBIF) [17, 18] can facilitate the adoption of PET protocols requiring input functions. In brain studies, IDIF is usually deduced from the dynamic images of the carotid arteries and hence is susceptible to partial volume effect [15–17]. Previous studies showed the feasibility of PBIF as a robust alternative to IDIF for some radiopharmaceuticals [17, 18]. PBIF is generated by averaging the normalized patient-specific arterial input functions (PSAIFs) deduced from a cohort of subjects. Several normalization techniques have been reported in the literature, for example, traditional scaling using blood samples by correlating the measured plasma activity with the AUC [17]; correlation of the PBIF with PSAIF venous samples [19]; scaling by injected dose and weight [20, 21]; and non-invasive scaling using individual parameters like weight, body surface area (BSA), and lean body mass (LBM) [17]. Many studies have assessed the feasibility of PBIF for kinetic analysis using [^18^F]FDG [18, 22–25], yet very few studies involved neuroreceptor PET tracers [17, 26], including TSPO brain studies [19, 21, 27]. To the best of our knowledge, no PET kinetic modeling study has been performed with [^11^C]DPA-713 using PBIF.

The main aim of this study is to assess the feasibility of using PBIF instead of the patient-specific AIF for [^11^C]DPA-713 PET kinetic modeling. This was done by first estimating the test-retest repeatability of the [^11^C]DPA-713 PET imaging in healthy subjects. Based on the test-retest results, we then assessed the reproducibility of kinetic analysis of [^11^C]DPA-713 dynamic PET images of the brain with PBIF compared to PSAIF in healthy and PD subjects. The effect of PSAIF normalization techniques on the PBIF-based kinetic results was also investigated.

## Materials and methods

### Subjects

In total, twelve subjects (9 males and 3 females; age 56.6 ± 11.9 years) were recruited from a Parkinson’s disease (PD) dynamic [^11^C]DPA-713 PET research study. Six additional healthy male subjects (age 42.6 ± 11.2 years) were also included, out of which five healthy subjects underwent test-retest studies to assess the repeatability of DPA kinetics. The inclusion criteria for the PD cohort are PD clinical diagnosis of 3 to 12 years of duration from onset of symptoms, age 30 to 70 years at time of enrollment, Hoehn and Yahr stages 2–3, and absence of a clinical diagnosis of dementia. Exclusion criteria included subjects receiving dopamine receptor blocking agents or treatment with acetylcholinesterase inhibitors, history of another significant neurological or major psychiatric disorder, or autoimmune disorders within the past 5 years. For screening purpose, all patients including healthy volunteers had a blood sample (3 mL) collected for TSPO (rs6971) genotype analysis. Three different genotypes are defined: low-affinity binders (LAB), mixed-affinity binders (MAB), and high-affinity binders (HAB). Patients that are low-affinity binders were excluded from participation. Detailed information on all subjects is shown in Supplementary Table S1.

### PET measurements and reconstruction

For the PET studies, 526.4 ± 73.6 MBq (14.2 ±1.9 mCi) of [^11^C]DPA-713 was administered through bolus-intravenous injection, followed by flushing 10–15 ml of saline solution. PET data were acquired simultaneously after injection in list-mode format on a 4-ring Siemens Biograph mCT^TM^ for a total of 90 min. The PET data were reconstructed into 32 dynamic frames (6×10 s, 4×30 s, 3×60 s, 2×120 s, 5×240 s, 12×300 s) using ordered subset expectation maximization (OSEM) with attenuation, scatter, and randoms corrections. Continuous arterial sampling was performed at 15-s intervals for the first 10 min using an automated fraction collector, followed by five additional samples collected at 20, 30, 45, 60, and 90 min respectively. Each of the blood samples was weighed and counted using a Wizard® automatic gamma counter (Perkin Elmer), and then, the activity concentration was calculated. Blood samples drawn at 5, 10, 20, 30, 45, 60, and 90 min post-injection were also used to estimate metabolite fractions using the HPLC method of analysis. The blood time activity curves (TACs) were finally corrected for metabolites, yielding a metabolite-corrected, arterial input function.

### Data analysis and kinetic modeling

Each subject underwent a T1-weighted MRI scan. Inter-frame head motion correction was achieved by rigidly co-registering the individual dynamic PET frames to the last 10 min image set using PMOD (version 3.8; PMOD Technologies Ltd). The resulting dynamic image set was then rigidly registered to the T1-MR image set. Brain regions were delineated on the MRI images using the FreeSurfer software [28], the corresponding volumes of interest (VOIs) were overlaid on the co-registered and motion-corrected dynamic PET images, and finally, the corresponding TACs were deduced.

Kinetic modeling was done for each patient using the Logan *V*_T_ model [29]:

1 $$ \frac{\int_0^tC(T) dT}{C(t)}={V}_T\frac{\int_0^t{C}_p(T) dT}{C(t)}+\mathrm{constant}\ \left(t\ge {t}^{\ast}\right) $$

where *C*(*t*) is the regional time activity curves (TACs), *C*_*p*_ is the input function, and *t*^∗^ is the time at which the plot of $\frac{\int_0^tC(T) dT}{C(t)}$ versus $\frac{\int_0^t{C}_p(T) dT}{C(t)}$ reaches linearity. The linearity time was determined using the maximum admissible error criterion as described by Ichise et al. [30]. This automatically searches for the minimum time after which the relative error of every data point in the Logan plot is lower than the given error threshold. A 10% error criteria was used in this study as also suggested by similar TSPO studies [27]. An example of the Logan fit for a sample patient using both PSAIF and PBIF is shown in Supplementary Figure S7.

Kinetic analysis was performed using the PSAIFs and then repeated using the PBIFs. For each of the selected brain structures (white matter, cerebellum, thalamus, caudate, putamen, pallidum, brainstem, hippocampus, and amygdala), the total volume of distribution (*V*_*T*_) was estimated with the blood volume fixed to 5%. These brain regions were selected mainly because they show great affinity for [^11^C]DPA-713 binding.

### Test-retest repeatability and reliability

Five healthy volunteers underwent a test-retest within the same day to assess the reproducibility of the kinetic parameters in the brain structures. Kinetic analysis was carried out for all the selected brain regions, for both the test and retest datasets, using the Logan *V*_*T*_ model and the corresponding PSAIF’s. The repeatability of *V*_*T*_ was assessed using the Bland-Altman analysis [31]:

2 $$ \% Relative\ Diff,D=\frac{Retest- Test}{\raisebox{1ex}{$\left( Retest+ Test\right)$}\!\left/ \!\raisebox{-1ex}{$2$}\right.}\times 100 $$

3 $$ Mean\ Bias=\frac{\sum_{n=1}^ND}{N} $$

where *N*= number of subjects

The corresponding 95% limits of agreement (LOA) and the coefficient of repeatability (CR) between test and retest were determined using:

4 $$ LOA= Mean\ Bias\pm 1.96 SD $$

5 $$ CR=1.96\times \sqrt{\frac{\sigma^2}{N-1}} $$

where *σ*^2^ is the variance of the relative difference, *D*, between the test and retest estimates. This represents the value below which the relative difference between test and retest is expected to lie with a 95% probability [31, 32].

### Generation of population-based input functions

The individual PSAIFs were fitted using the “tri-exponential” function and then corrected for metabolites after fitting the later using “Watabe” function (as incorporated in PMOD). The PSAIFs were also fitted with “bi-exponential and gamma” function, but the tri-exponential function gave the best fit for all subjects involved in this study. Sample graphs of the fitted PSAIF, metabolite fraction, and the resulting metabolite-corrected PSAIF are shown in Supplementary Figure S1.

The PBIFs were generated from the metabolite-corrected PSAIFs of all the 18 subjects under review (samples in Supplementary Figure S5A). The metabolite-corrected PSAIFs for all subjects were interpolated to the same time grid (with a step of 1 s), and then, their peaks were aligned to the 30 s time point where the majority of the IF peaks occurred. In order to reduce the influence of subject-induced variation on the generated PBIF, each of the metabolite-corrected PSAIFs was normalized separately by three methods: (a) Weight_subject_× Dose_Injected_, (b) the corresponding AUC, and (c) Weight_subject_×AUC.

For each subject, PBIF was generated by averaging the normalized PSAIF of the other 17 subjects—leave-one-out procedure [17, 20, 33, 34]. Individual subject IFs were then generated by appropriately scaling the PBIF with the corresponding factor, i.e., (a) Weight_subject_×Dose_Injected_, (b) the corresponding AUC, and (c) Weight_subject_×AUC.

Since the normalized PBIF does not have arterial blood samples, the AUC scaling was done by tail-fitting the normalized PBIF and the PSAIF using the time points 30, 45, 60, and 90 min. Additionally, three pseudo-time points (37.5, 52.5, and 75 min) were created as the average of PSAIF at 30 and 45 min, 45 and 60 min, and 60 and 90 min respectively. This was done in order to find an optimal time point which minimizes the difference between the original PSAIF AUC and the PBIF AUC obtained by scaling with one (or two) late blood sample, following a similar approach for TSPO study [27].

The reproducibility of *V*_*T*_ using PBIF was assessed using Bland-Altman analysis, with PSAIF values as gold reference. For each structure, the % relative difference (Relative Diff), *D*, between the parameters was estimated using:

6 $$ \% Relative\ Diff,D=\frac{P_{\mathrm{PBIF}}-{P}_{\mathrm{PSAIF}}}{P_{\mathrm{PSAIF}}}\times 100 $$

where *P*_PBIF_ and *P*_PSAIF_ are the kinetic parameters generated by PBIF and PSAIF respectively.

The bias and the corresponding 95% upper and lower LOA were estimated using Eqs. (3 and 4).

### Statistical analysis

Data were analyzed using the SPSS (IBM SPSS statistics for windows, version 26.0) and Real statistics (http://www.real-statistics.com/) software. Normality of distribution was tested using the Shapiro-Wilk test. The statistical difference between the three normalization techniques was evaluated using the one-way analysis of variance (ANOVA). The pairwise *t*-test was also performed as a follow-up test to ANOVA in order to reveal which specific pair of the normalization techniques is significantly different, and Bonferroni correction was applied to correct for the potential error due to multiple testing. In all cases, a *P*-value < 0.05 was considered to suggest statistical significance.

**Fig. 1:**
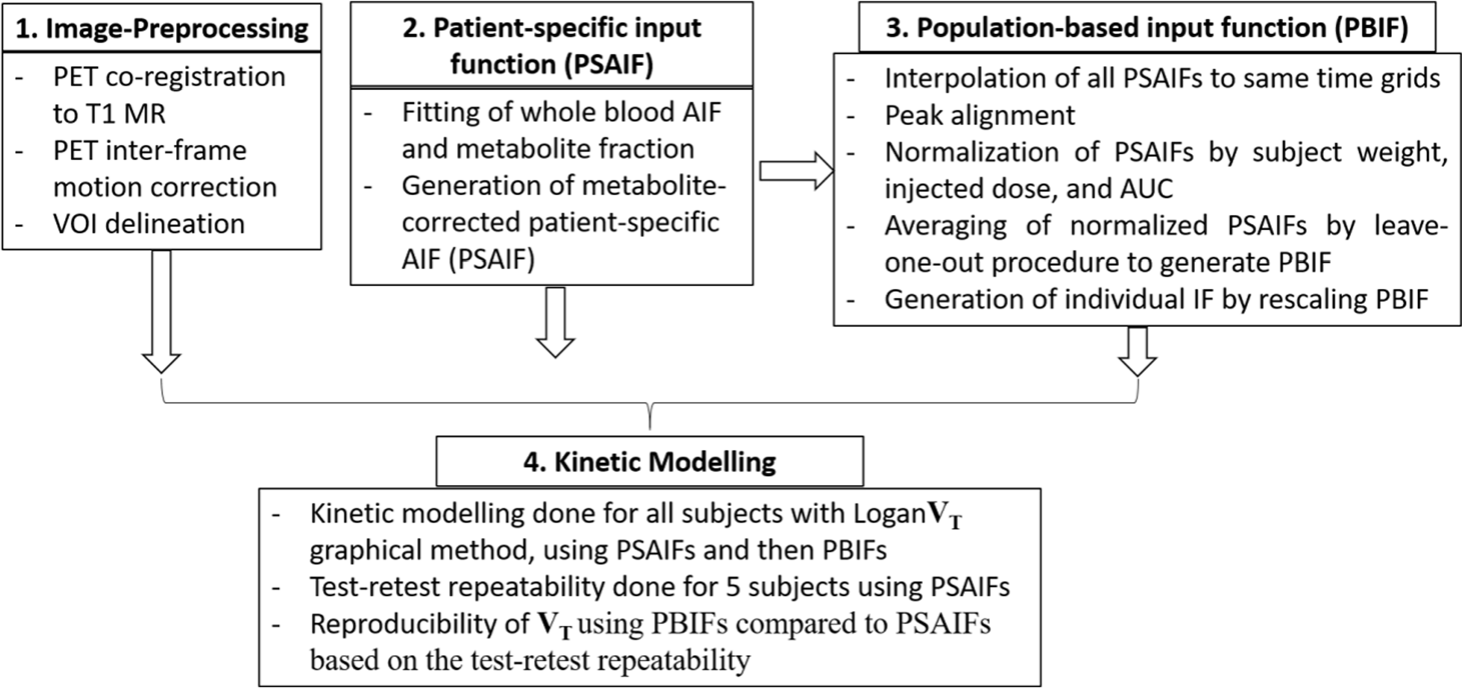
The workflow chart showing the major steps involved in this study

The major steps involved in this study are represented with a workflow chart in Fig. 1.

## Results

### Test-retest repeatability and reliability

**Fig. 2:**
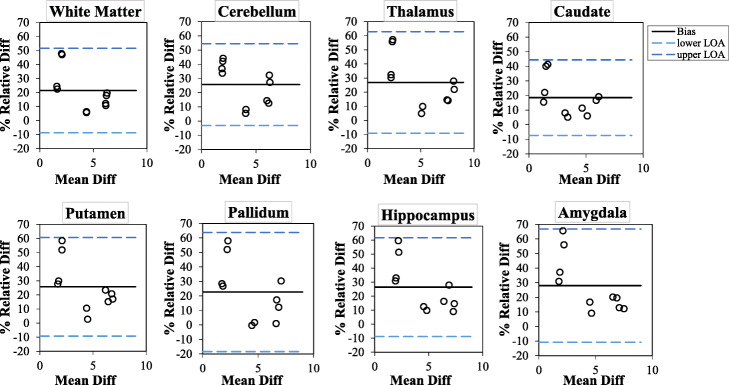
Bland-Altman plot comparing the test-retest repeatability of *V*_T_ estimates for all selected brain regions of interest (the datapoints are from both left and right hemispheres). The solid line is the mean % bias between test and retest *V*_T_ estimate, while the dashed lines represent the %LOA

The repeatability of the *V*_T_ estimates for all selected brain regions of interest in the test-retest studies are shown in Table 1 and Fig. 2. For all the brain regions of interest, the mean of the *V*_T_ estimates from all the healthy volunteers is between 3.18 and 4.91 for test estimates and 3.68 and 5.92 for the retest. The *V*_T_ estimates in the retest studies exhibited positive bias (ranging from 20 to 30%) compared to those deduced from the test studies. A systematic bias is also noticed between the test and the retest results, where all the differences lie above the zero line. The 95% LOA lies within ~3% and ~70% for all regions.

**Table 1:** Bland-Altman analysis of the variation in *V*_T_ estimates between the test and retest

Regions	Test mean	Retest mean	Mean % bias ± SD	95% LOA	ICC
White matter	3.74 ± 2.02	4.43 ± 2.14	21.43 ± 15.08	−8.74 to 51.60	0.93
Cerebellum	3.61 ± 1.87	4.50 ± 2.15	25.69 ± 14.40	−3.12 to 54.50	0.88
Thalamus	4.51 ± 2.50	5.61 ± 2.77	26.89 ± 18.01	−9.12 to 62.90	0.90
Caudate	3.16 ± 1.84	3.68 ± 2.02	18.52 ± 12.94	−7.36 to 44.40	0.95
Putamen	3.83 ± 2.12	4.71 ± 2.31	25.71 ± 17.47	−9.24 to 60.65	0.91
Pallidum	4.06 ± 2.24	4.83 ± 2.35	22.70 ± 20.56	−18.41 to 63.82	0.91
Brainstem	4.91 ± 2.92	5.92 ± 3.15	23.61 ± 15.80	−7.99 to 55.21	0.94
Hippocampus	4.09 ± 2.29	5.04 ± 2.39	26.44 ± 17.65	−8.85 to 61.73	0.91
Amygdala	4.04 ± 2.35	4.99 ± 2.42	27.98 ± 19.40	−10.81 to 66.78	0.92

### Comparison between PSAIF and PBIF

**Fig. 3:**
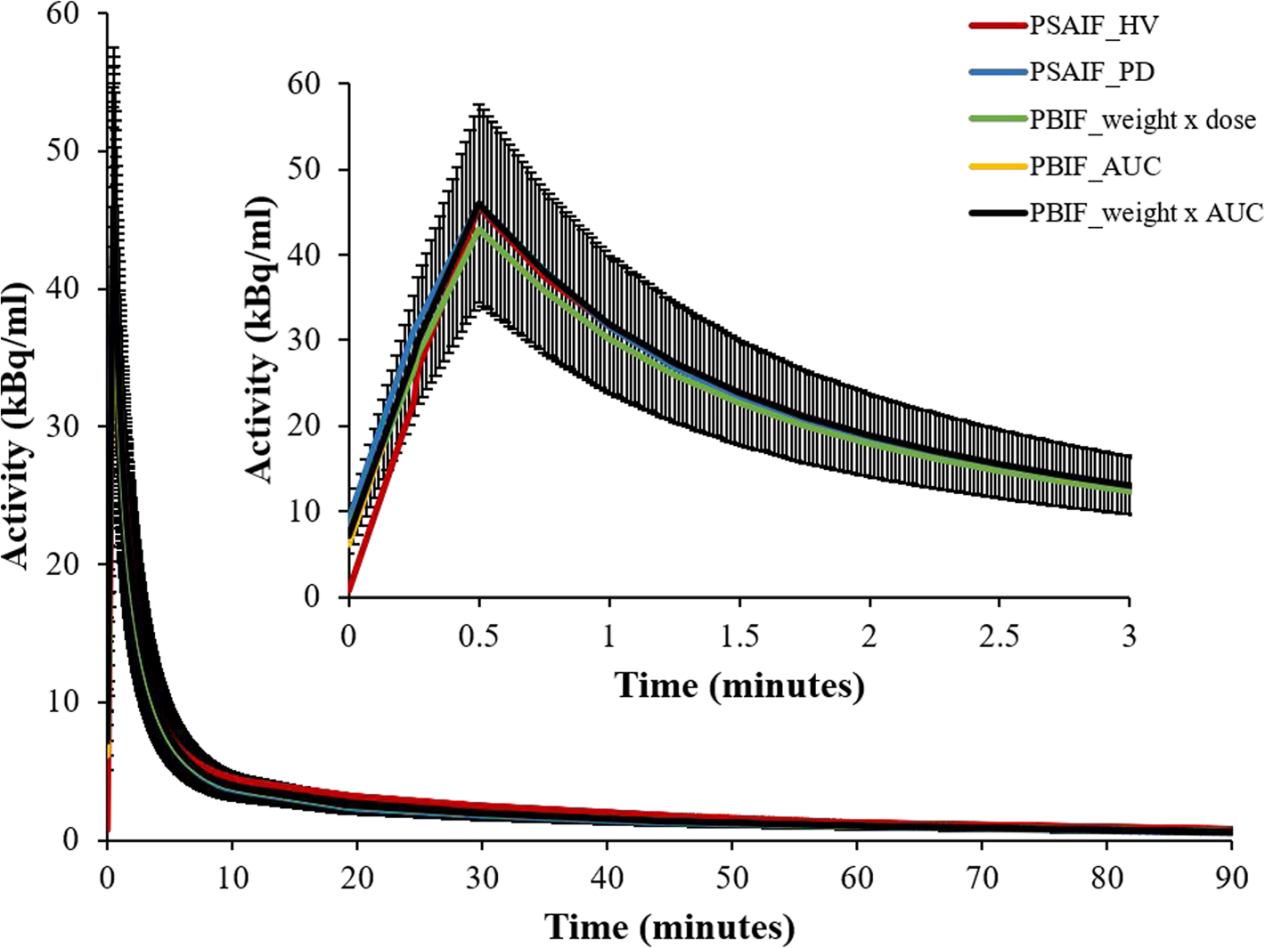
Comparison between the averaged PSAIFs of HV and PD subjects, and the resulting PBIF generated from the three normalization techniques. The inner plot shows the zoomed IF over the first 3 min. The standard deviation (SD) is shown for the PBIF generated by normalization with Weight_subject_×AUC.

Before generating the PBIF from the pool of subjects, we first examined the shape of the PSAIFs between groups (HV versus PD) and genotype (MAB vs HAB). The results are shown in Fig. 2 and Supplementary S2. Visual inspection of the average IF for HV and PD showed no difference between groups (Fig. 3). Also, the log-transformation plot showed no difference in the peak or tail for different groups and genotype (Supplementary Figure S2).

**Fig. 4:**
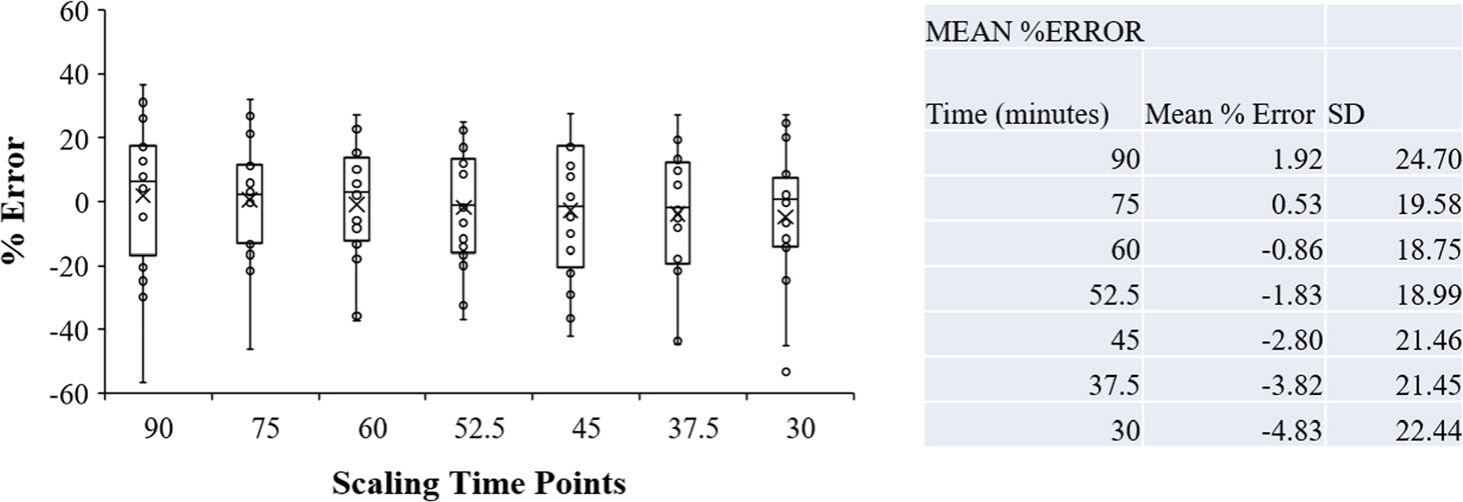
AUC comparison of the scaled PBIF using the different time points and the original PSAIF. Note that no blood was collected at time points 37.5, 52.5, and 75 min; they were just average time points of 30 and 45 min, 45 and 60 min, and 60 and 90 min respectively

Therefore, the PSAIFs for all the eighteen subjects involved in this study were pooled together to generate the PBIF using the three normalization techniques used in this study. The individual IFs were generated by appropriately scaling the PBIF with the corresponding normalization factor. AUC scaling was done by tail-fitting the normalized PBIF and the PSAIF using the time points 30, 37.5, 45, 52.5, 60, 75, and 90 min. The AUC of the scaled PBIF using the different time points and the original PSAIF were then compared by evaluating the %error (result in Fig. 4). Although there is no significant difference in the %error between the different time points, the sample at 75 min yielded the least %error of 0.53%. Since 75 min was actually an average between the blood collected at 60 and 90 min, the AUC scaling (i.e., scaling with one (or two) late blood sample) in this study was done by tail-fitting the PBIF and the PSAIF using the last 30 min time points (i.e., between 60 and 90 min).

### Evaluation of the PBIF and the normalization criteria

**Fig. 5:**
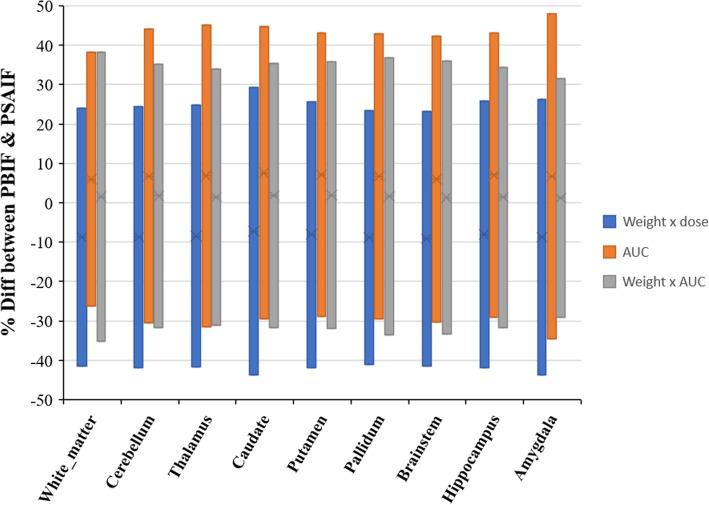
The % relative difference in *V*_T_ and the LOAs between PSAIF and PBIF of some specific structures as generated by the three normalization techniques

Figure 5 shows the % difference and the LOAs between the *V*_T_ estimates generated by the PSAIF and PBIF for selected brain regions. The comparison is made using PSAIF and the PBIF generated by the three normalization techniques. The mean % difference is −10% for Weight_subject_×Dose_Injected_, +8% for AUC, and +2% for Weight_subject_×AUC, while the LOAs lie within ±45% for Weight_subject_×Dose_Injected_, ±50% for AUC, and ±38% for Weight_subject_×AUC. The ANOVA analysis shows a significant difference between the results generated by the three normalization techniques for all brain structures except the brainstem (*P*-value = 0.095). Although for the same brainstem, the pairwise test shows a significant difference between Weight_subject_×Dose_Injected_ versus AUC (*P*-value = 0.034). In all cases, normalizing by Weight_subject_×AUC yielded the smallest % bias and variability (% bias = ±2%; LOA = ±38% for all brain regions).

**Fig. 6:**
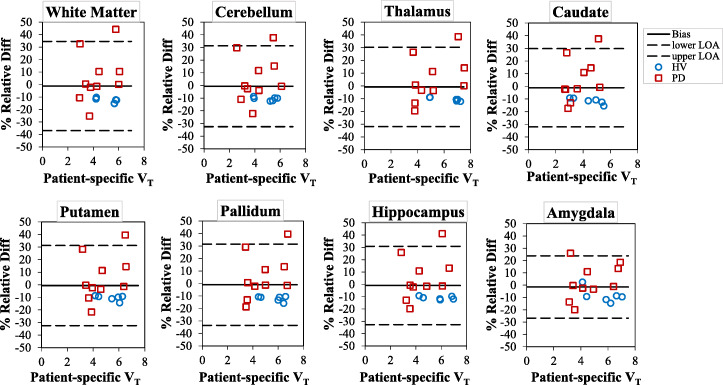
Bland-Altman analysis showing the variation in the *V*_T_ between the PSAIF and PBIF (normalization with Weight_subject_×AUC). The values are shown for the HV (blue circles) and the PD (red boxes) groups. The solid line is the mean % bias between PSAIF and PBIF *V*_T_ estimate, while the dashed lines represent the %LOA

The mean bias (±SD) between the PSAIF and PBIF for the *V*_T_ generated by normalization with Weight_subject_×AUC are shown in Fig. 6 and Table 2 (for all brain regions).

**Table 2:** Bland-Altman analysis of the variation in *V*_T_ estimate between the PSAIF and PBIF (normalization with Weight_subject_×AUC)

Regions	Mean % bias ± SD	95% LOA
White matter	1.51 ± 18.74	−35.22 to 38.23
Cerebellum	1.73 ± 17.09	−31.75 to 35.22
Thalamus	1.45 ± 16.57	−31.02 to 33.93
Caudate	1.80 ± 17.14	−31.79 to 35.40
Putamen	1.91 ± 17.27	−31.96 to 35.77
Pallidum	1.57 ± 17.95	−33.60 to 36.75
Brainstem	1.28 ± 17.67	−33.35 to 35.92
Hippocampus	1.36 ± 16.86	−31.67 to 34.41
Amygdala	1.21 ± 15.42	−29.01 to 31.45

The mean bias for *V*_T_ lies within ±2%, with amygdala showing the smallest (1.21%) deviation and putamen showing the highest (1.91%). Overall, the 95% LOA for all brain regions lies within ±38%.

We also evaluated the reproducibility of the *V*_T_ generated with PBIF to that of PSAIF using the estimated t*, and other goodness of fit criteria (AIC, *R*^2^, and % standard error (SE)). The result is shown in Supplementary Figure S8. There is no difference between the *V*_T_s generated by PBIF and PSAIF based on these criteria.

### Agreement of *V*_T_ between PSAIF and PBIF (based on disease groups and genotype)

**Fig. 7:**
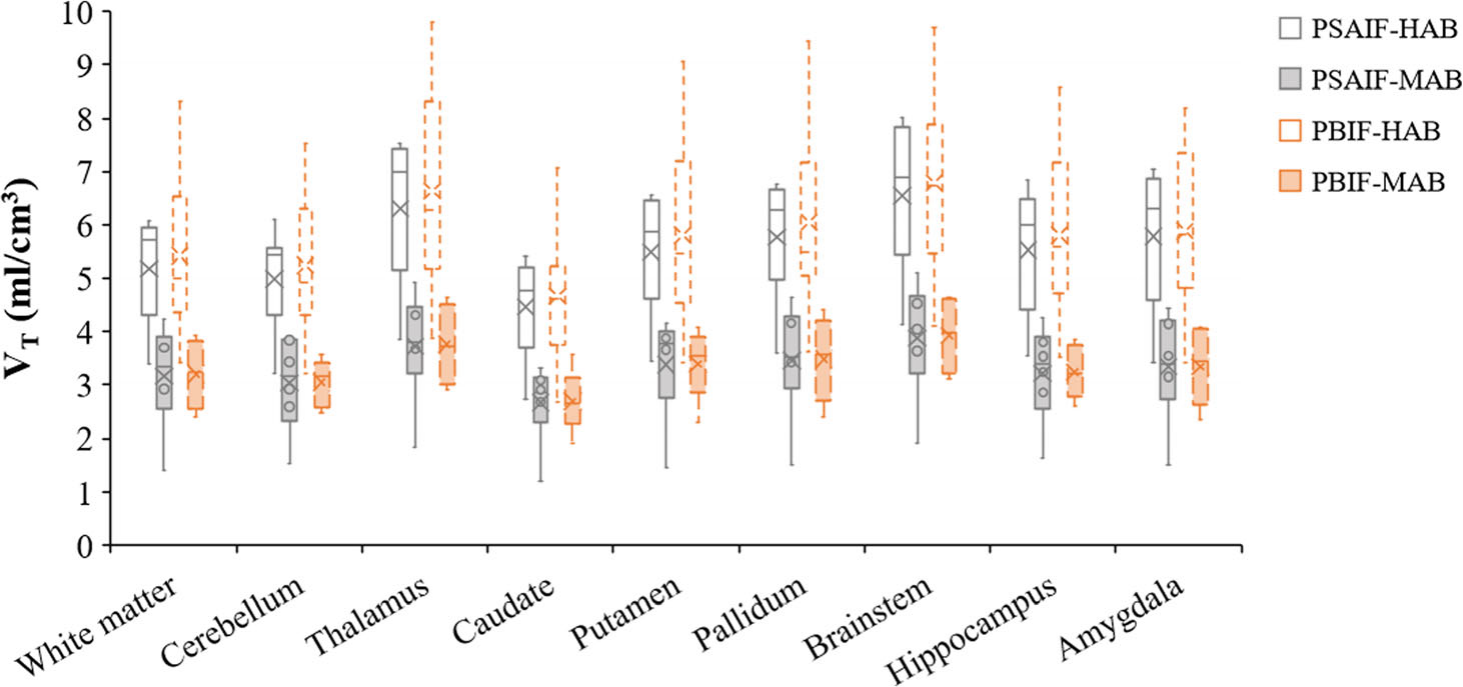
Logan *V*_T_ values for all subjects calculated with PSAIF and PBIF for the two genotype groups (HAB and MAB). Comparing MAB to HAB, there is a significant reduction in *V*_T_ both with PSAIF and PBIF. PSAIF showed an average reduction of 40% in *V*_T_ across the brain regions, while the average reduction with PBIF is 42%

Finally, we estimated how well the PBIF-kinetics replicates the PSAIF-kinetics based on disease groups (HV versus PD) and genotype (MAB versus HAB). Figure 7 shows the Logan *V*_T_ values for all subjects calculated with PSAIF and PBIF for the two genotype groups (HAB and MAB). For all brain regions, the average *V*_T_ values obtained from PBIF is slightly higher than PSAIF, but the difference is not significant for each genotype group (%difference = 4.53%, *P*-value = 0.73 for HAB; and %difference = 0.73%, *P*-value = 0.96 for MAB). Comparing MAB to HAB, there is a significant reduction in *V*_T_ both with PSAIF and PBIF. PSAIF showed an average reduction of 40% in *V*_T_ across the brain regions, while the average reduction with PBIF is 42%. *T*-test showed a *P*-value < 0.01 for all the brain regions, both for PSAIF and PBIF, and on average, the *P*-value for PBIF is about 50% higher than PSAIF.

**Fig. 8:**
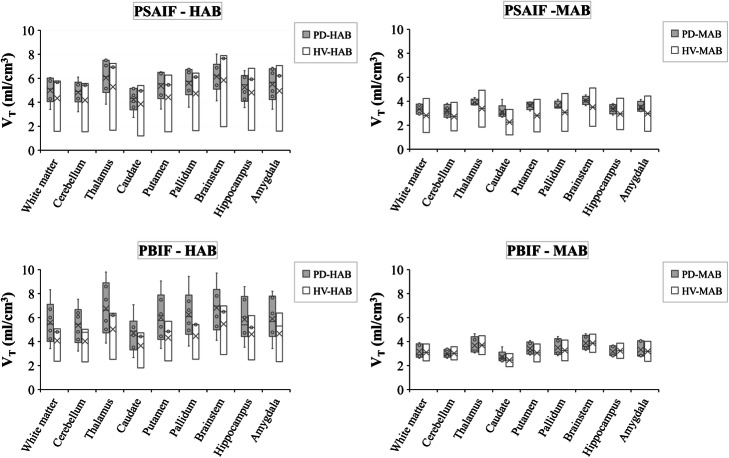
Logan *V*_T_ values between HV and PD subjects and also HAB and MAB groups calculated with PSAIF and PBIF

Figure 8 shows the difference in Logan *V*_T_ values calculated with PSAIF and PBIF between HV and PD subjects, and also HAB and MAB groups. The average *V*_T_ values are insignificantly higher in PD patients compared to HV. PBIF tend to overestimate the difference between PD and HV for HAB (%difference = 32.33%, *P*-value = 0.32 with PBIF; %difference = 13.28%, *P*-value = 0.64 with PSAIF). However, this difference is underestimated in MAB (%difference = 6.84%, *P*-value = 0.77 with PBIF; %difference = 20.92%, *P*-value = 0.55 with PSAIF).

## Discussion

Several studies have shown the feasibility to image neuroinflammation in multiple sclerosis (MS), Parkinson’s disease (PD), and Alzheimer’s disease (AD) using [^11^C]DPA-713 PET for quantifying differences between patients and controls [4, 9–12]. Accurate quantification of tracer uptake and metabolism in the tissue through kinetic modeling often requires blood sampling [13] or some alternative approach such as simplified reference modeling [35, 36], cluster analysis [35, 37], or image-derived input function (IDIF) techniques. The apparent limitations of these approaches [15–17, 19, 21, 38] are giving way to the exploration of the population-based input function (PBIF) approach as a more quantitatively reliable and less invasive alternative.

In this study, we have assessed the reproducibility of kinetic analysis of [^11^C]DPA-713 dynamic PET images using PBIF, compared to PSAIF, in a cohort of subjects with Parkinson’s disease and healthy volunteers. The major steps involved in this study are represented in a workflow chart in Fig. 1. The repeatability of the *V*_T_ estimate was also assessed in a cohort of healthy volunteers that underwent a test-retest [^11^C]DPA-713 dynamic PET within the same day. Kinetic analysis with PSAIF was determined to be reproducible with PBIF if the corresponding LOA are within those of the test-retest study.

### Test-retest repeatability

The test-retest repeatability study of the [^11^C]DPA-713 uptake exhibited systematic increase in uptake values between test and retest (Fig. 2 and supplementary Figure S3) although the cause of this systematic bias is yet to be fully explored in same day test-retest repeatability studies because most repeatability studies are done days or even weeks apart [39, 40]. Few recent studies that performed same day test-retest have reported the same systematic bias, and they suggested that the possible explanation to this systematic bias could be due to hormone-mediated changes in TSPO expression, tonic changes due to scan-related stress/anxiety, or alteration in blood cholesterol due to food intake between the test and retest scans [39, 41–43]. While performing test and retest studies under similar conditions on different days could eliminate this bias [40], other parameters such as alteration in TSPO density due to chronic disease as well as non-disease-related factors may be difficult to control [43].

One potential approach to compensate for the systematic bias between the test and retest studies is by normalization by the corresponding kinetic parameters of the GM as suggested by past studies [39, 43–45] and also shown by this study (Supplementary Figure S3 and S4). Without GM normalization, the % relative difference between test and retest *V*_T_ values lies significantly above the zero line for all structures, indicating that retest values are always higher than test values. But with GM normalization, the % relative difference is symmetric about the zero line. The mean % Diff, the LOA and the CR are also significantly reduced, thereby improving repeatability. However, past studies involving gray matter normalization was validated in a clinical population (HIV with associated cognitive deficits) with regional inflammation. We believe there is no sufficient justification of using the GM normalization approach for PD cohorts since neuroinflammation can occur in any brain region, and therefore, we elected not to adopt it.

Another possible explanation for the high test-retest bias as reported in this study is the small regions of interest used and the relatively small number of healthy subjects (*n*=5) who underwent the test-retest scans. This is in agreement with a TSPO study from Jucaite et al. [39] which showed that the mean bias between test and retest was low in large brain regions (such as the whole brain, cortical gray matter and white matter) but high in smaller brain regions. They also attributed the large variability to the limited sample size, and this was also confirmed by Plaven-Sigray et al. [46] who estimated a test-retest variability in six healthy controls and obtained a variability of about 21% in *V*_T_. In fact, Collste et al. [40] carried out a test-retest study with six subjects examined on the same day and other six subjects examined 2–5 days apart. The % bias was within 14–27% for subjects examined within the same day and 0.2–8.4% for subjects examined 2–5 days apart. We therefore recommend that more research still needs to be done in order to fully understand the effect of small sample size, small brain regions of interest, as well as days between test and retest scans on the test-retest reproducibility.

### Generation and evaluation of the PBIF

The PBIF was generated from the PSAIF of all 18 subjects after examining the shape of the PSAIFs between groups (HV versus PD) and genotype (MAB vs HAB). This was motivated by Owen et al. [47, 48], who demonstrated that the second generation TSPO tracers target two binding sites in humans, which leads to three affinity patterns: low-, high-, and mixed-affinity binders (LABs, HABs, and MABs respectively). Past researches have shown that this variability in binding affinity has a major influence on the kinetic parameters where the values for HABs could be approximately twice that of MABs [48, 49]. For [^11^C]-DPA-713 dynamic PET studies, Coughlin et al. [43] argued that those genotypes as well as other unknown physiological factors have varying degrees of influence on the global TSPO changes in the brain, thereby hindering accurate PET analysis, even among individuals with the same genotype. This was also confirmed in other TSPO studies [39, 45, 50]. Our results (Fig. 3 and Supplementary Figure S2) however showed no significant difference in PSAIF between the groups, and so, all subject PSAIFs were included in the generation of the PBIF. This was also in agreement with other studies [27, 51].

Ye et al. [52] opined that the bias in kinetic parameter estimation in direct reconstruction with PBIF depends on the normalization and scaling technique used. In this study, we have assessed and compared three normalization approaches: (a) Weight_subject_×Dose_Injected_, (b) AUC, and (c) Weight_subject_×AUC. These normalization techniques were chosen because they reduced the influence of subject-induced variation on the generated PBIF. We also normalized the PSAIFs by different methods (as shown in supplementary Figure S6 and Table S2). However, since our aim is to reduce patient variability in the generation of the PBIF, we decided to go with the above stated normalization techniques. An example of the normalized PSIFs and the resulting PBIF are shown in Supplementary Figure S5. The performance of these techniques was evaluated using the percent relative difference between the PSAIF- and PBIF-derived *V*_T_ in selected brain regions (Fig. 5). There is a significant difference between the three normalization techniques for all brain structures except the brainstem.

Several normalization techniques have been reported in the literature which include traditional scaling using blood samples by correlating the measured plasma activity at a given time-point with the AUC [17]; correlation of the PBIF with AIF at any time-point using venous samples [19]; by accounting for injected dose and weight [20, 21]; non-invasive scaling using individual parameters like weight, body surface area (BSA), and lean body mass (LBM) [17]. In this study, we have assessed the three aforementioned normalization approaches. Subsequently, a subject IF was deduced by scaling the PBIF by his/her weight and injected dose. In the case of AUC normalization, this was measured after scaling the PBIF by the ratio of the average activity concentration of blood samples acquired over the last 30 min of the dynamic scan (i.e., between 60 and 90 min) and that of the tail of the PBIF over the same time frames. This setting was used as this best minimizes the error between PSAIF AUC and the scaled PBIF (Fig. 4).

Precisely, the AUC between PSAIF and PBIF was minimized by scaling the PBIF with an arterial blood value at 75 min, as also recommended by past similar TSPO studies [27, 51]. It has been shown that venous blood samples may practically be used instead for scaling purpose since arterial and venous blood tend to reach equilibrium at about 30–45 min post-injection time [17]. Although this was not tested in this work, but similar TSPO studies have also found that PBIF can be appropriately scaled using one blood sample [27, 51]. Since the utmost aim is to potentially alleviate the need for arterial blood sampling, the AUC component of the PBIF normalization can be obtained by scaling the normalized PBIF by the ratio of the average activity concentration of blood samples (possibly venous blood) acquired over the last 30 min of the dynamic scan (i.e., between 60 and 90 min) and that of the tail of the PBIF over the same time frames (as was done in this study). However, more relevant clinical studies need to be conducted to establish a correlation between activity concentration in arterial and venous blood samples at these latter time points of the scan.

In this study, normalization by Weight_subject_×AUC yielded the smallest % bias (**±**2%) and variability (LOAs **±**38%) between PBIF and PSAIF (Fig. 5). *V*_T_ measured with PBIF showed good reproducibility (LOA of ±38%) but with a positive bias (±2%) (Fig. 6 and Table 2). These were also in agreement with the findings of Lavisse et al. [19]. As a final note, the reproducibility of the PBIF-based *V*_T_ estimates compared with PSAIF-based *V*_T_ fall well within the test-retest results (Table 1), hence showing the feasibility of [^11^C]-DPA-713 PET kinetic modeling using PBIF.

PBIF was able to reproduce the PSAIF kinetic results because of the similar patterns in average AIF between disease groups and genotypes (Fig. 3 and Supplementary Figure S2). Although we expect that PBIF cannot exactly reproduce the peak and shape of the PSAIF (as shown in Supplementary Figure S2), but having a similar AUC between PSAIF and PBIF will result in less bias in kinetic parameter estimation. That is why previous studies have recommended that Logan *V*_T_ method is more suitable for PBIF than 2-tissue compartment model because Logan *V*_T_ relies on the AUC of the IF and therefore less sensitive to the shape [20, 53].

A major limitation for this study is the relatively small sample size (*n* = 18), even though our findings are in agreement with previous results of smaller (*n* = 9) [19] and larger (*n* = 42) [20] sample sizes. A common factor among these studies is the normalization of the individual input functions to remove variabilities in the PBIF. This might suggest that the efficiency of the PBIF in accurately estimating the kinetic parameters depends less on the sample size used but more on the normalization. This was also consolidated by Ye et al. [52] who opined that the bias in kinetic parameter estimation in direct reconstruction with PBIF was mostly due to inaccuracy in normalization and scaling.

## Conclusion

This study demonstrated the feasibility of [^11^C]-DPA-713 PET kinetic modeling using PBIF with Logan graphical analysis, thus potentially alleviating the need for arterial blood sampling. Moreover, it was shown that the optimal result in terms of kinetic parameter accuracy was obtained when the PSAIFs were normalized with Weight_subject_×AUC.

## Supplementary Information

**Additional file 1: Table S1**. Demographic information of all the PD patients and healthy volunteers (HV) included in this study. **Figure S1**. Sample graphs of the fitted PSAIF, metabolite fraction and the resulting metabolite corrected PSAIF. **Figure S2**. Comparison between the PSAIF of healthy volunteers (HV) and Parkinson’s disease (PD) patients based on genotype. Log transformation was applied on the plot A, and it shows the average PSAIF for each group. Plots B and C show the box plots for the individual subjects in each group. The results demonstrate no substantial differences in the peak and the tail of the IFs across the groups. Therefore, all IFs were used to estimate the PBIF (shown in black). **Figure S3**. Bland-Altman plot comparing the test-retest repeatability of Vt estimates for all selected brain regions of interest: (A) without GM normalization and (B) with GM normalization respectively. The solid line is the mean % bias between test and retest V_T_ estimate, while the doted and dashed lines represent the %CI and %LOA respectively. **Figure S4**. Overlaid normalized PSAIFs from all 18 patients (A) and the resulting PBIF generated by normalization with Weight_subject_×AUC (B). The zoomed PBIF over the first 5 minutes is also shown. In (b), the blue points are the mean PBIF while the red points are the standard error of the mean (SEM). **Figure S5**. Example of the Logan VT plot generated by the patient-specific input function (upper row) and the population-based input function (lower row). **Figure S6**. Comparing the PBIF-estimated V_T_ and PSAIF-estimated VT using the goodness of fit criteria.

## References

[1] Cumming P, Burgher B, Patkar O, Breakspear M, Vasdev N, Thomas P, Liu GJ, Banati R (2018). Sifting through the surfeit of neuroinflammation tracers. J Cereb Blood Flow Metab.

[2] Hagens M, Berckel B, Barkhof F (2016). Novel MRI and PET markers of neuroinflammation in multiple sclerosis. Curr Opin Neurol.

[3] Airas L, Rissanen E, Rinne J (2017). Imaging of microglial activation in MS using PET: research use and potential future clinical application. Mult Scler.

[4] Boutin H, Chauveau F, Thominiaux C, Gregoire MC, James ML, Trebossen R, Hantraye P, Dolle F, Tavitian B, Kassiou M (2007). C-11-DPA-713: a novel peripheral benzodiazepine receptor PET ligand for in vivo imaging of neuroinflammation. J Nucl Med.

[5] Fookes CJR, Pham TQ, Mattner F, Greguric I, Loc’h C, Liu X, Berghofer P, Shepherd R, Gregoire MC, Katsifis A (2008). Synthesis and biological evaluation of substituted F-18 imidazo 1,2-a pyridines and F-18 pyrazolo 1,5-a pyrimidines for the study of the peripheral benzodiazepine receptor using positron emission tomography. J Med Chem.

[6] Kreisl WC, Fujita M, Fujimura Y, Kimura N, Jenko KJ, Kannan P, Hong J, Morse CL, Zoghbi SS, Gladding RL, Jacobson S, Oh U, Pike VW, Innis RB (2010). Comparison of [(11)C]-(R)-PK 11195 and [(11)C]PBR28, two radioligands for translocator protein (18 kDa) in human and monkey: implications for positron emission tomographic imaging of this inflammation biomarker. Neuroimage..

[7] James ML, Fulton RR, Vercoullie J, Henderson DJ, Garreau L, Chalon S, Dolle F, Selleri S, Guilloteau D, Kassiou M (2008). DPA-714, a new translocator protein-specific ligand: synthesis, radiofluorination, and pharmacologic characterization. J Nucl Med.

[8] Endres CJ, Pomper MG, James M, Uzuner O, Hammoud DA, Watkins CC, Reynolds A, Hilton J, Dannals RF, Kassiou M (2009). Initial evaluation of C-11-DPA-713, a novel TSPO PET ligand, in humans. J Nucl Med.

[9] Doorduin J, Klein HC, Dierckx RA, James M, Kassiou M, de Vries EFJ (2009). C-11 -DPA-713 and F-18 -DPA-714 as new PET tracers for TSPO: a comparison with C-11 -(R)-PK11195 in a rat model of herpes encephalitis. Mol Imaging Biol.

[10] Rosenberg P, Endres C, Lyketsos C, Coughlin J, Kassiou M, Pomper M (2011). Quantifying translocator protein (TSPO) in Alzheimer’s disease and cognitively healthy older persons with 11C-DPA-713 PET imaging. Alzheimers Dement.

[11] Zimmer ER, Leuzy A, Benedet AL, Breitner J, Gauthier S, Rosa-Neto P (2014). Tracking neuroinflammation in Alzheimer’s disease: the role of positron emission tomography imaging. J Neuroinflammation.

[12] Terada T, Yokokura M, Yoshikawa E, Futatsubashi M, Kono S, Konishi T, Miyajima H, Hashizume T, Ouchi Y (2016). Extrastriatal spreading of microglial activation in Parkinson’s disease: a positron emission tomography study. Ann Nucl Med.

[13] Bentourika M (2006). Kinetic modeling of PET-FDG in the brain without blood sampling. Comput Med Imaging Graph.

[14] Kang Y, Mozley PD, Verma A, Schlyer D, Henchcliffe C, Gauthier SA, Chiao PC, He B, Nikolopoulou A, Logan J, Sullivan JM, Pryor KO, Hesterman J, Kothari PJ, Vallabhajosula S (2018). Noninvasive PK11195-PET Image analysis techniques can detect abnormal cerebral, microglial activation in Parkinson’s disease. J Neuroimaging.

[15] Watabe H, Channing MA, Riddell C, Jousse F, Libutti SK, Carrasquillo JA (2001). Noninvasive estimation of the aorta input function for measurement of tumor blood flow with. IEEE Trans Med Imaging.

[16] Mourik JEM, van Velden FHP, Lubberink M, Kloet RW, van Berckel BNM, Lammertsma AA, Boellaard R (2008). Image derived input functions for dynamic High Resolution Research Tomograph PET brain studies. Neuroimage..

[17] Zanotti-Fregonara P, Hines CS, Zoghbi SS, Liow JS, Zhang Y, Pike VW, Drevets WC, Mallinger AG, Zarate CA, Fujita M, Innis RB (2012). Population-based input function and image-derived input function for C-11 (R)-rolipram PET imaging: methodology, validation and application to the study of major depressive disorder. Neuroimage..

[18] Brock CS, Young H, Osman S, Luthra SK, Jones T, Price PM (2005). Glucose metabolism in brain tumours can be estimated using [18F] 2-fluorodeoxyglucose positron emission tomography and a population-derived input function scaled using a single arterialised venous blood sample. Int J Oncol.

[19] Lavisse S, Garcia-Lorenzo D, Peyronneau MA, Bodini B, Thiriez C, Kuhnast B, Comtat C, Remy P, Stankoff B, Bottlaender M (2015). Optimized quantification of translocator protein radioligand F-18-DPA-714 uptake in the brain of genotyped healthy volunteers. J Nucl Med.

[20] Zanotti-Fregonara P, Hirvonen J, Lyoo CH, Zoghbi SS, Rallis-Frutos D, Huestis MA, Morse C, Pike VW, Innis RB (2013). Population-based input function modeling for F-18 FMPEP-d(2), an inverse agonist radioligand for cannabinoid CB1 receptors: validation in clinical studies. PLoS One.

[21] MacAskill MG, Walton T, Williams L, Morgan TEF, Alcaide-Corral CJ, Dweck MR (2019). Kinetic modelling and quantification bias in small animal PET studies with [18F]AB5186, a novel 18 kDa translocator protein radiotracer. PLoS One.

[22] Wakita K, Imahori Y, Ido T, Fujii R, Horii H, Shimizu M, Nakajima S, Mineura K, Nakamura T, Kanatsuna T (2000). Simplification for measuring input function of FDG PET: investigation of 1-point blood sampling method. J Nucl Med.

[23] Vriens D, de Geus-Oei L-F, Oyen WJG, Visser EP (2009). A curve-fitting approach to estimate the arterial plasma input function for the assessment of glucose metabolic rate and response to treatment. J Nucl Med.

[24] Tsuchida T, Sadato N, Yonekura Y, Nakamura S, Takahashi N, Sugimoto K, Waki A, Yamamoto K, Hayashi N, Ishii Y (1999). Noninvasive measurement of cerebral metabolic rate of glucose using standardized input function. J Nucl Med.

[25] Takikawa S, Dhawan V, Spetsieris P, Robeson W, Chaly T, Dahl R, Margouleff D, Eidelberg D (1993). Noninvasive quantitative fluorodeoxyglucose PET studies with an estimated input function derived from a population-based arterial blood curve. Radiology..

[26] Takikawa S, Dhawan V, Chaly T, Robeson W, Dahl R, Zanzi I, Mandel F, Spetsieris P, Eidelberg D (1994). Input functions for 6-[fluorine-18]fluorodopa quantitation in parkinsonism: comparative studies and clinical correlations. J Nucl Med.

[27] Mabrouk R, Strafella AP, Knezevic D, Ghadery C, Mizrahi R, Gharehgazlou A, Koshimori Y, Houle S, Rusjan P (2017). Feasibility study of TSPO quantification with [18F]FEPPA using population-based input function. PLoS One.

[28] Fischl B (2012). FreeSurfer. Neuroimage.

[29] Logan J, Fowler JS, Volkow ND, Wolf AP, Dewey SL, Schlyer DJ, MacGregor RR, Hitzemann R, Bendriem B, Gatley SJ, Christman DR (1990). Graphical analysis of reversible radioligand binding from time-activity measurements applied to [N-11C-methyl]-(-)-cocaine PET studies in human subjects. J Cereb Blood Flow Metab.

[30] Ichise M, Toyama H, Innis RB, Carson RE (2002). Strategies to improve neuroreceptor parameter estimation by linear regression analysis. J Cereb Blood Flow Metab.

[31] Bland JM, Altman DG (1986). Statistical methods for assessing agreement between two methods of clinical measurement. Lancet..

[32] Schwartz J, Humm JL, Gonen M, Kalaigian H, Schoder H, Larson SM, Nehmeh SA (2011). Repeatability of SUV measurements in serial PET. Med Phys.

[33] Karakatsanis N, Zhou Y, Lodge M, Casey M, Wahl R, Subramanian R (2015). Clinical whole-body PET Patlak imaging 60-90min post-injection employing a population-based input function. J Nucl Med.

[34] Meyer PT, Circiumaru V, Cardi CA, Thomas DH, Bal H, Acton PD (2006). Simplified quantification of small animal [18F]FDG PET studies using a standard arterial input function. Eur J Nucl Med Mol Imaging.

[35] Arlicot N, Vercouillie J, Ribeiro MJ, Tauber C, Venel Y, Baulieu JL, Maia S, Corcia P, Stabin MG, Reynolds A, Kassiou M, Guilloteau D (2012). Initial evaluation in healthy humans of F-18 DPA-714, a potential PET biomarker for neuroinflammation. Nucl Med Biol.

[36] Golla SSV, Boellaard R, Oikonen V, Hoffmann A, van Berckel BNM, Windhorst AD, Virta J, Haaparanta-Solin M, Luoto P, Savisto N, Solin O, Valencia R, Thiele A, Eriksson J, Schuit RC, Lammertsma AA, Rinne JO (2015). Quantification of F-18 DPA-714 binding in the human brain: initial studies in healthy controls and Alzheimer’s disease patients. J Cereb Blood Flow Metab.

[37] Ribeiro M-J, Vercouillie J, Debiais S, Cottier J-P, Bonnaud I, Camus V, Banister S, Kassiou M, Arlicot N, Guilloteau D (2014). Could 18 F-DPA-714 PET imaging be interesting to use in the early post-stroke period?. EJNMMI Res.

[38] Hoekstra CJ, Hoekstra OS, Lammertsma AA (1999). On the use of image-derived input functions in oncological fluorine-18 fluorodeoxyglucose positron emission tomography studies. Eur J Nucl Med.

[39] Jučaite A, Cselényi Z, Arvidsson A, Åhlberg G, Julin P, Varnäs K, Stenkrona P, Andersson J, Halldin C, Farde L (2012). Kinetic analysis and test-retest variability of the radioligand [11C](R)-PK11195 binding to TSPO in the human brain - a PET study in control subjects. EJNMMI Res.

[40] Collste K, Forsberg A, Varrone A, Amini N, Aeinehband S, Yakushev I, Halldin C, Farde L, Cervenka S (2016). Test-retest reproducibility of [(11)C]PBR28 binding to TSPO in healthy control subjects. Eur J Nucl Med Mol Imaging.

[41] Drugan RC (1996). Peripheral benzodiazepine receptors: molecular pharmacology to possible physiological significance in stress-induced hypertension. Clin Neuropharmacol.

[42] Gavish M, Bachman I, Shoukrun R, Katz Y, Veenman L, Weisinger G, Weizman A (1999). Enigma of the peripheral benzodiazepine receptor. Pharmacol Rev.

[43] Coughlin JM, Wang Y, Ma S, Yue C, Kim PK, Adams AV, Roosa HV, Gage KL, Stathis M, Rais R, Rojas C, McGlothan JL, Watkins CC, Sacktor N, Guilarte TR, Zhou Y, Sawa A, Slusher BS, Caffo B, Kassiou M, Endres CJ, Pomper MG (2014). Regional brain distribution of translocator protein using [11C]DPA-713 PET in individuals infected with HIV. J Neurovirol.

[44] Herranz E, Giannì C, Louapre C, Treaba CA, Govindarajan ST, Ouellette R, Loggia ML, Sloane JA, Madigan N, Izquierdo-Garcia D, Ward N, Mangeat G, Granberg T, Klawiter EC, Catana C, Hooker JM, Taylor N, Ionete C, Kinkel RP, Mainero C (2016). Neuroinflammatory component of gray matter pathology in multiple sclerosis. Ann Neurol.

[45] Vera JH, Guo Q, Cole JH, Boasso A, Greathead L, Kelleher P, Rabiner EA, Kalk N, Bishop C, Gunn RN, Matthews PM, Winston A (2016). Neuroinflammation in treated HIV-positive individuals. Neurology..

[46] Plavén-Sigray P, Matheson GJ, Cselényi Z, Jucaite A, Farde L, Cervenka S (2018). Test-retest reliability and convergent validity of (R)-[11C]PK11195 outcome measures without arterial input function. EJNMMI Res.

[47] Owen DRJ, Gunn RN, Rabiner EA, Bennacef I, Fujita M, Kreisl WC, Innis RB, Pike VW, Reynolds R, Matthews PM, Parker CA (2011). Mixed-affinity binding in humans with 18-kDa translocator protein ligands. J Nucl Med.

[48] Owen DR, Guo Q, Kalk NJ, Colasanti A, Kalogiannopoulou D, Dimber R, Lewis YL, Libri V, Barletta J, Ramada-Magalhaes J, Kamalakaran A, Nutt DJ, Passchier J, Matthews PM, Gunn RN, Rabiner EA (2014). Determination of [(11)C]PBR28 binding potential in vivo: a first human TSPO blocking study. J Cereb Blood Flow Metab.

[49] Hagens MHJ, Golla SV, Wijburg MT, Yaqub M, Heijtel D, Steenwijk MD, Schober P, Brevé JJP, Schuit RC, Reekie TA, Kassiou M, van Dam AM, Windhorst AD, Killestein J, Barkhof F, van Berckel BNM, Lammertsma AA (2018). In vivo assessment of neuroinflammation in progressive multiple sclerosis: a proof of concept study with F-18 DPA714 PET. J Neuroinflammation.

[50] Wang Y, Coughlin J, Zhou Y, Ma S, Endres C, Pomper M (2013). A method for personalized brain mapping of neuroinflammation using 11C-DPA-713 PET. J Nucl Med.

[51] Buchert R, Dirks M, Schütze C, Wilke F, Mamach M, Wirries A-K, Pflugrad H, Hamann L, Langer LBN, Wetzel C, Lukacevic M, Polyak A, Kessler M, Petrusch C, Bengel FM, Geworski L, Rupprecht R, Weissenborn K, Ross TL, Berding G (2020). Reliable quantification of (18)F-GE-180 PET neuroinflammation studies using an individually scaled population-based input function or late tissue-to-blood ratio. Eur J Nucl Med Mol Imaging.

[52] Ye Q, Lyu Z, Yao S, Dong Y, Liu H, Wu J, et al. Direct 4D Patlak reconstruction in dynamic FDG PET imaging with population-based input function. In: 2018 IEEE Nuclear Science Symposium and Medical Imaging Conference, NSS/MIC 2018 - Proceedings. 2018. p. 1–4.

[53] Rissanen E, Tuisku J, Luoto P, Arponen E, Johansson J, Oikonen V, Parkkola R, Airas L, Rinne JO (2015). Automated reference region extraction and population-based input function for brain C-11 TMSX PET image analyses. J Cereb Blood Flow Metab.

