# Supplementary material for: Population-based input function for TSPO quantification and kinetic modeling with [11C]-DPA-713
Source: EJNMMI Phys. 2021 Apr 29;8:39. doi: 10.1186/s40658-021-00381-8 (PMC8085191; doi:10.1186/s40658-021-00381-8)
Supplement: Supplementary file 1 — Additional file 1: Table S1. Demographic information of all the PD patients and healthy volunteers (HV) included in this study. Figure S1. Sample graphs of the fitted PSAIF, metabolite fraction and the resulting metabolite corrected PSAIF. Figure S2. Comparison between the PSAIF of healthy volunteers (HV) and Parkinson’s disease (PD) patients based on genotype. Log transformation was applied on the plot A, and it shows the average PSAIF for each group. Plots B and C show the box plots for the individual subjects in each group. The results demonstrate no substantial differences in the peak and the tail of the IFs across the groups. Therefore, all IFs were used to estimate the PBIF (shown in black). Figure S3. Bland-Altman plot comparing the test-retest repeatability of Vt estimates for all selected brain regions of interest: (A) without GM normalization and (B) with GM normalization respectively. The solid line is the mean % bias between test and retest VT estimate, while the doted and dashed lines represent the %CI and %LOA respectively. Figure S4. Overlaid normalized PSAIFs from all 18 patients (A) and the resulting PBIF generated by normalization with Weightsubject×AUC (B). The zoomed PBIF over the first 5 minutes is also shown. In (b), the blue points are the mean PBIF while the red points are the standard error of the mean (SEM). Figure S5. Example of the Logan VT plot generated by the patient-specific input function (upper row) and the population-based input function (lower row). Figure S6. Comparing the PBIF-estimated VT and PSAIF-estimated VT using the goodness of fit criteria. [file 40658_2021_381_MOESM1_ESM.docx]

**SUPPLEMENTARY MATERIAL**

**Population-Based Input Function for TSPO quantification and Kinetic Modeling with [^11^C]-DPA-713**

1. **Demographic and Genotype Information of all Subjects involved in the Study**

***Table S1:*** *Demographic information of all the PD patients and healthy volunteers (HV) included in this study*

| **Patient ID** | | **Gender** | **Age (years)** | | **Weight (Kg)** | | **Height (m)** | **BMI** | **Group** | | **Genotype** | |
| --- | --- | --- | --- | --- | --- | --- | --- | --- | --- | --- | --- | --- |
| S1 | | male | 67 | | 80.38 | | 1.70 | 27.81 | PD | | MAB | |
| S2 | | male | 58 | | 86.80 | | 1.78 | 27.40 | PD | | HAB | |
| S3 | | male | 62 | | 75.00 | | 1.80 | 23.15 | PD | | MAB | |
| S4 | | male | 70 | | 65.30 | | 1.75 | 21.26 | PD | | HAB | |
| S5 | | male | 61 | | 56.79 | | 1.78 | 17.98 | PD | | HAB | |
| S6 | | male | 62 | | 73.60 | | 1.72 | 24.88 | PD | | HAB | |
| S7 | | female | 54 | | 61.90 | | 1.61 | 23.79 | PD | | MAB | |
| S8 | | male | 67 | | 78.00 | | 1.78 | 24.62 | PD | | MAB | |
| S9 | | male | 67 | | 83.91 | | 1.73 | 28.13 | PD | | HAB | |
| S10 | | female | 64 | | 72.57 | | 1.78 | 22.96 | PD | | HAB | |
| S11 | | female | 60 | | 47.17 | | 1.60 | 18.42 | PD | | HAB | |
| S12 | | male | 62 | | 102.30 | | 1.63 | 38.71 | PD | | MAB | |
| S13 | | male | 58 | | 79.38 | | 1.80 | 24.50 | HV | | MAB | |
| S14 | | male | 50 | | 81.65 | | 1.68 | 28.93 | HV | | HAB | |
| S15 | | male | 28 | | 77.11 | | 1.85 | 22.53 | HV | | HAB | |
| S16 | | male | 38 | | 63.50 | | 1.68 | 22.50 | HV | | HAB | |
| S17 | | male | 41 | | 84.82 | | 1.75 | 27.70 | HV | | MAB | |
| S18 | | male | 48 | | 78.93 | | 1.63 | 29.71 | HV | | HAB | |
| **Average** | |  | **56.50** | | **74.95** | | **1.72** | **25.28** |  | |  | |
| **SD** | |  | **11.41** | | **12.59** | | **0.07** | **4.75** |  | |  | |
|  |  | | |  | |  | |  | |  | |  |
|  | **Number (male, female)** | | | **Average Age (years)** | | **Average weight (Kg)** | | **Average height (m)** | | **Average BMI** | |  |
| PD-MAB | 5 (4, 1) | | | 62.40 ± 5.32 | | 79.52 ± 14.60 | | 1.70 ± 0.09 | | 27.60 ± 6.46 | |  |
| PD-HAB | 7 (5, 2) | | | 63.3 ± 4.55 | | 68.8 ± 15.43 | | 1.74 ± 0.07 | | 22.70 ± 4.33 | |  |
| HV-MAB | 2 (2) | | | 49.5 ± 12.02 | | 82.1 ± 3.85 | | 1.78 ± 0.04 | | 26.10 ± 2.26 | |  |
| HV-HAB | 4 (4) | | | 41 ± 10.13 | | 75.3 ± 8.08 | | 1.71 ± 0.10 | | 25.92 ± 3.94 | |  |

1. **Fitting of Input Function and Metabolite Fraction**

We tried to fit the PSAIFs with “bi-exponential + gamma” and tri-exponential functions, but the tri-exponential function gave the best fit for all subjects involved in this study (An example is shown in Figure S1A where the tri-exponential fit best reproduced the blood data in terms of the peak and goodness of fit).

So, the individual PSAIFs were fitted using the “tri-exponential” function and then corrected for metabolites after fitting the later using “Watabe” function (as incorporated in PMOD). Sample graphs of the fitted PSAIF, metabolite fraction and the resulting metabolite corrected PSAIF are shown in Supplementary Figure S1

**
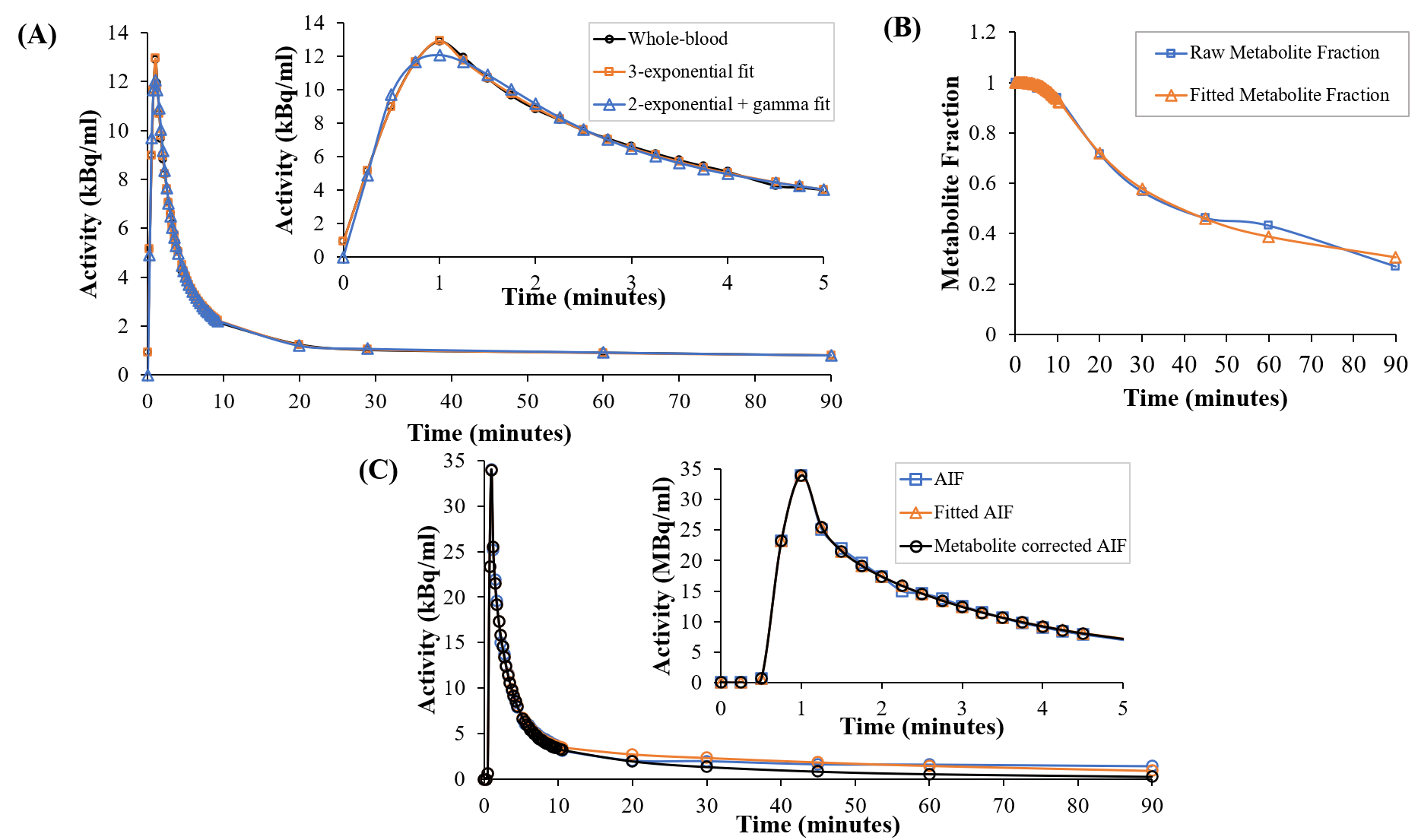
**

***Figure S1:*** *Sample graphs of the fitted PSAIF, metabolite fraction and the resulting metabolite corrected PSAIF*

1. **Comparison of input functions between HV and PD subjects based on genotype**


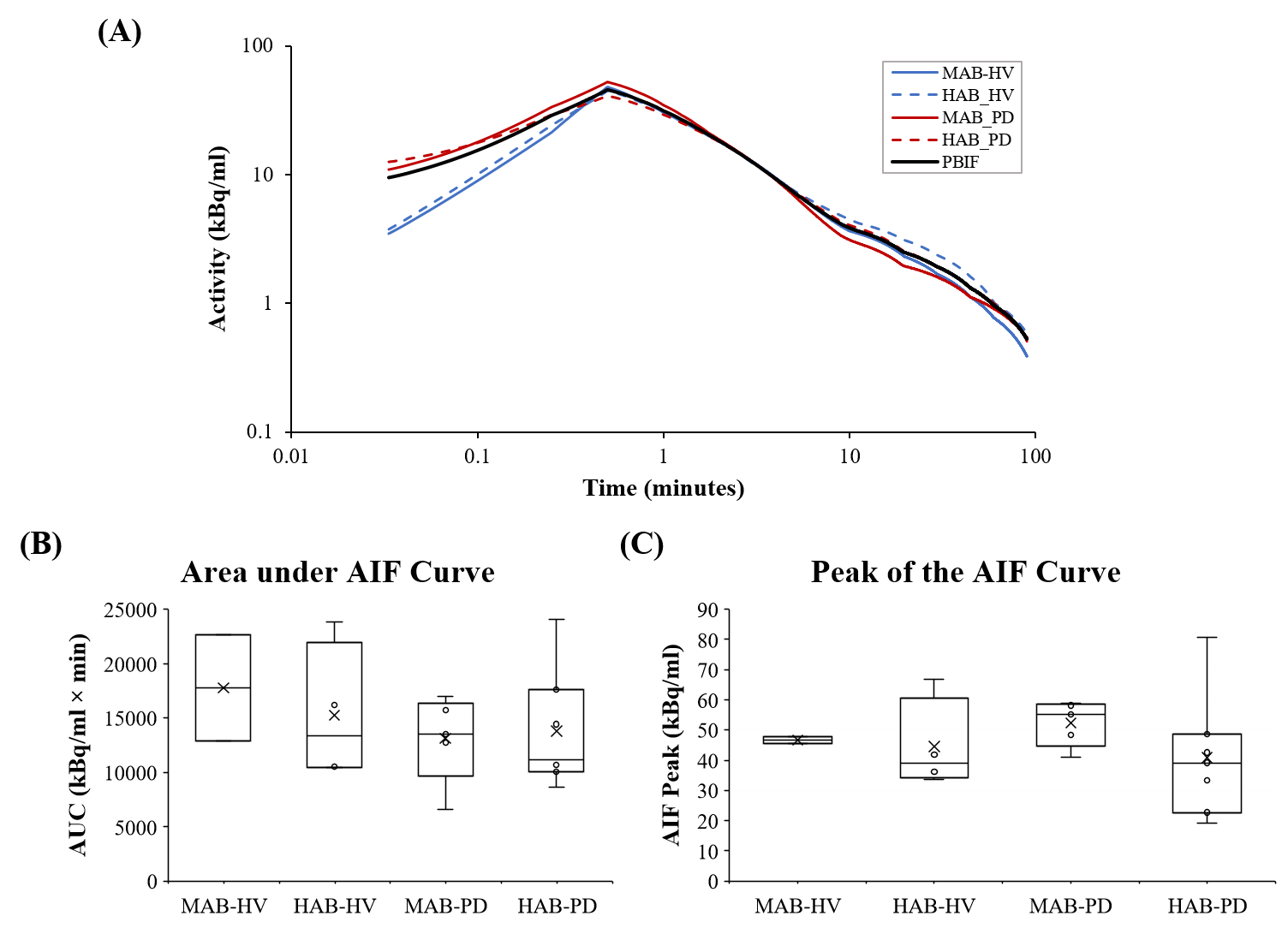


**Figure S2:** Comparison between the PSAIF of healthy volunteers (HV) and Parkinson’s disease (PD) patients based on genotype. Log transformation was applied on the plot A, and it shows the average PSAIF for each group. Plots B and C show the box plots for the individual subjects in each group. The results demonstrate no substantial differences in the peak and the tail of the IFs across the groups. Therefore, all IFs were used to estimate the PBIF (shown in black).

1. **Test-retest repeatability and Importance of Gray Matter (GM) Normalization**

The V_T_ estimates in the retest studies exhibited positive bias (ranging from 20 to 30%) compared to those deduced from the test studies (Figure S3A), where all the differences lie above the zero line (systematic bias). This systematic bias between test and retest V_T_ estimates could potentially be compensated for by normalization with the corresponding values of the GM, resulting in a bias within ±5% for all brain regions (Figure S3B). The LOA also reduced with GM normalization (from ~70% to ~20%).

Normalization by the GM also resulted in reducing both the % bias and CR between the test and retest kinetic parameters. The corresponding results with and without normalization by the GM are summarized in Figure S4. V_T_ values exhibited a large reduction in the mean % bias between test and retest studies after normalization by the corresponding GM values (from ~30% to ~5%). The V_T_ values also showed improved CR after GM normalization (from ~15% to ~5%) (Figure S4B).


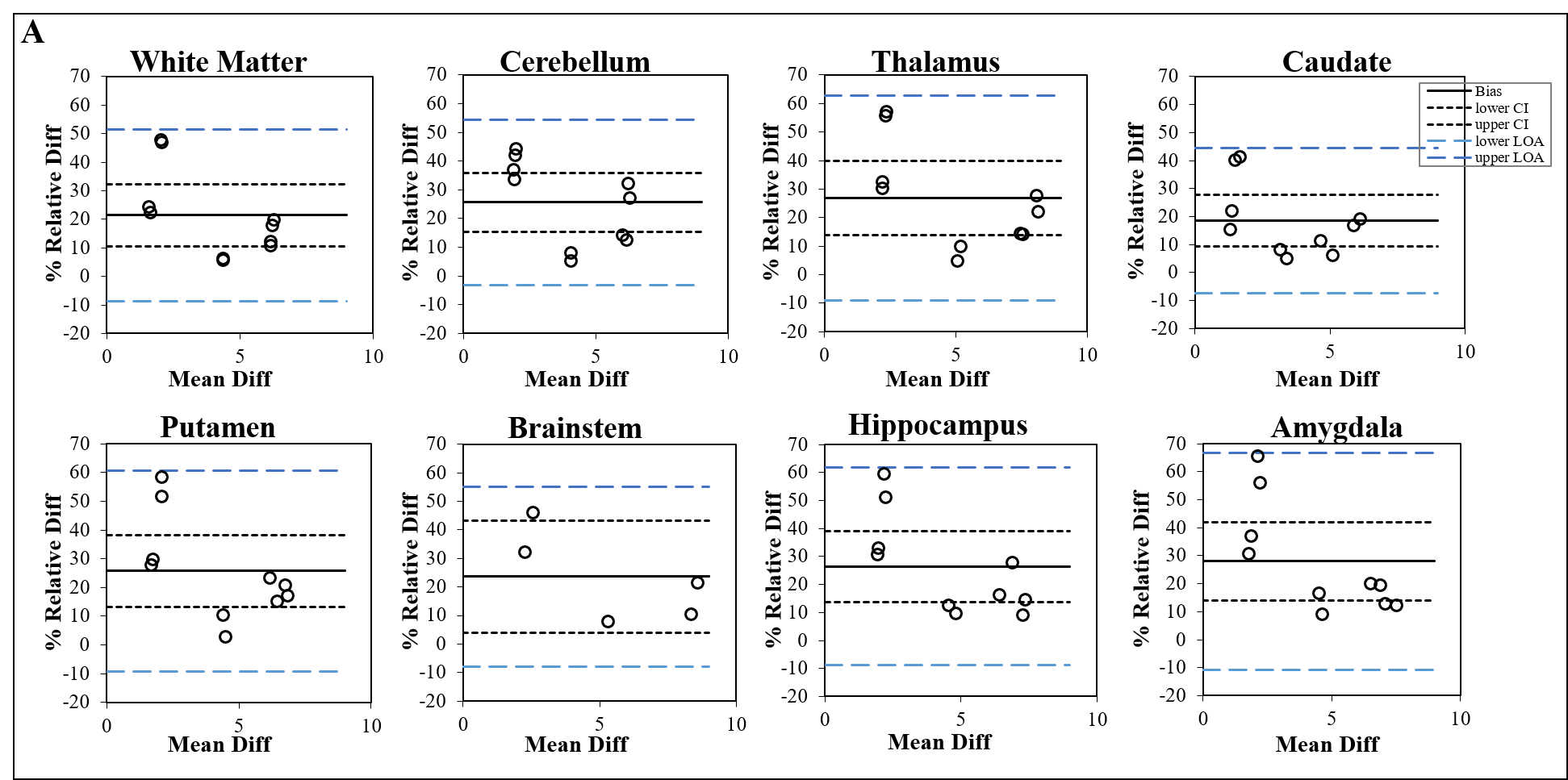


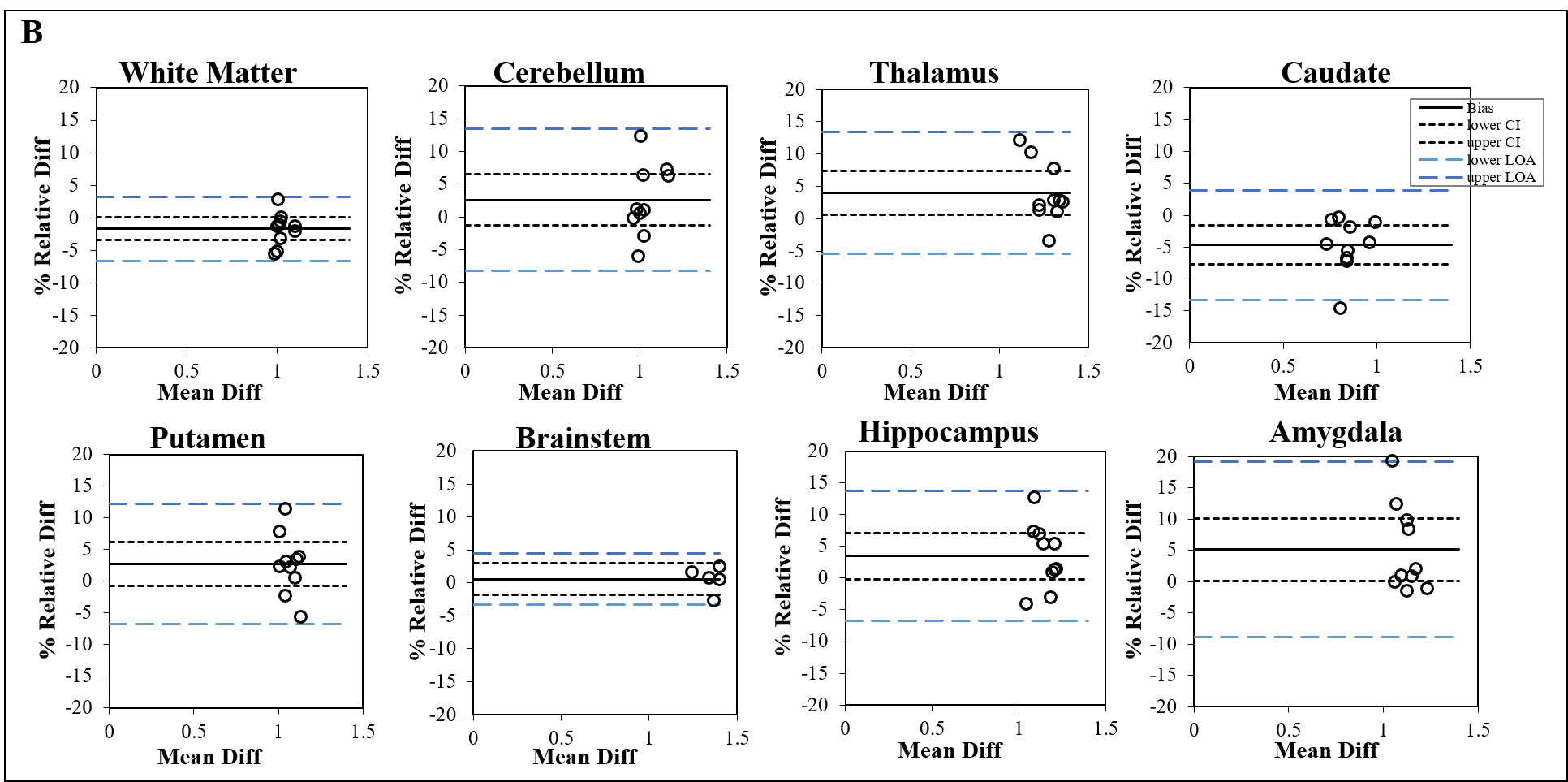


***Figure S3*** *Bland-Altman plot comparing the test-retest repeatability of Vt estimates for all selected brain regions of interest: (A) without GM normalization and (B) with GM normalization respectively. The solid line is the mean % bias between test and retest V_T_ estimate, while the doted and dashed lines represent the %CI and %LOA respectively*


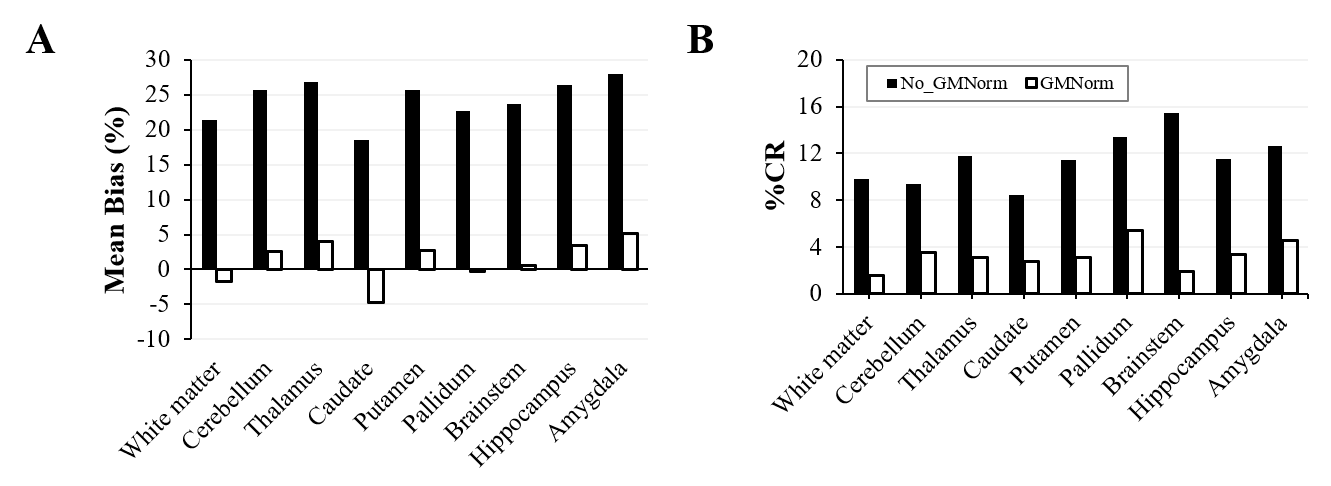


***Figure S4*** *Effect of GM normalization on the (A) mean % bias and (B) CR of the test-retest V_T_ estimate*

1. **Generation of PBIF and Evaluation of Normalization Technique**

In order to reduce the influence of subject-induced variation on the generated PBIF, each of the metabolite-corrected PSAIFs was normalized separately by three methods: (a) Weight_subject_$\times$ Dose_Injected_ (b) the corresponding AUC, and (c) Weight_subject_$\times$AUC, yielding three PBIFs for the study. Figure S4 shows the overlaid normalized PSAIFs (Figure S5A) and the resulting PBIF generated after normalizing each PSAIF by Weight_subject_$\times$AUC (Figure S5B).

*
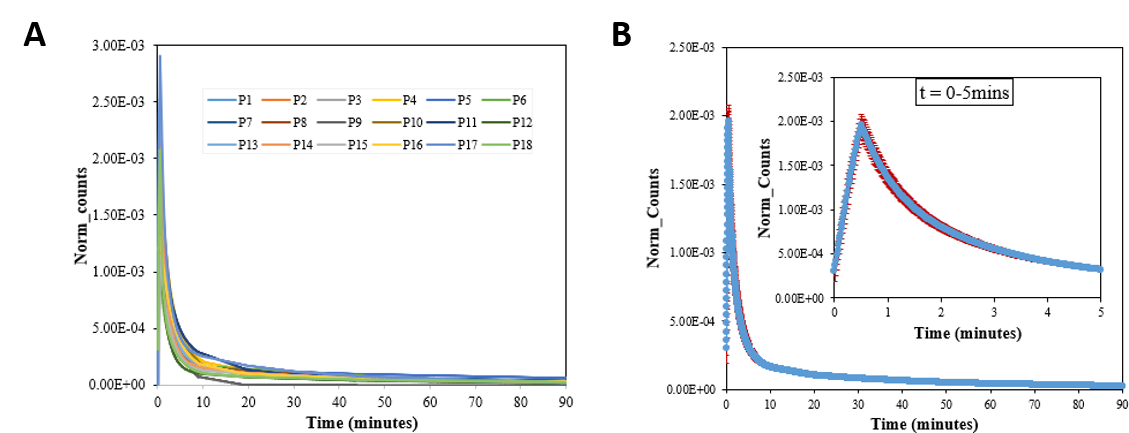
*

***Figure S5*** *Overlaid normalized PSAIFs from all 18 patients (A) and the resulting PBIF generated by normalization with* Weight_subject_$\times$AUC *(B). The zoomed PBIF over the first 5 minutes is also shown. In (b), the blue points are the mean PBIF while the red points are the standard error of the mean (SEM)*

We also carried out the scaling by Dose_Injected_ / Weight_subject_ as well as AUC / Weight_subject_. However, better results were obtained with injected dose $\times$ body weight and AUC $\times$ Weight_subject_ in terms of the patient variability and V_T_ reproducibility. Since our aim is to reduce patient variability in the generation of the PBIF (as stated in the Method section), we decided to go with the above stated normalization route.


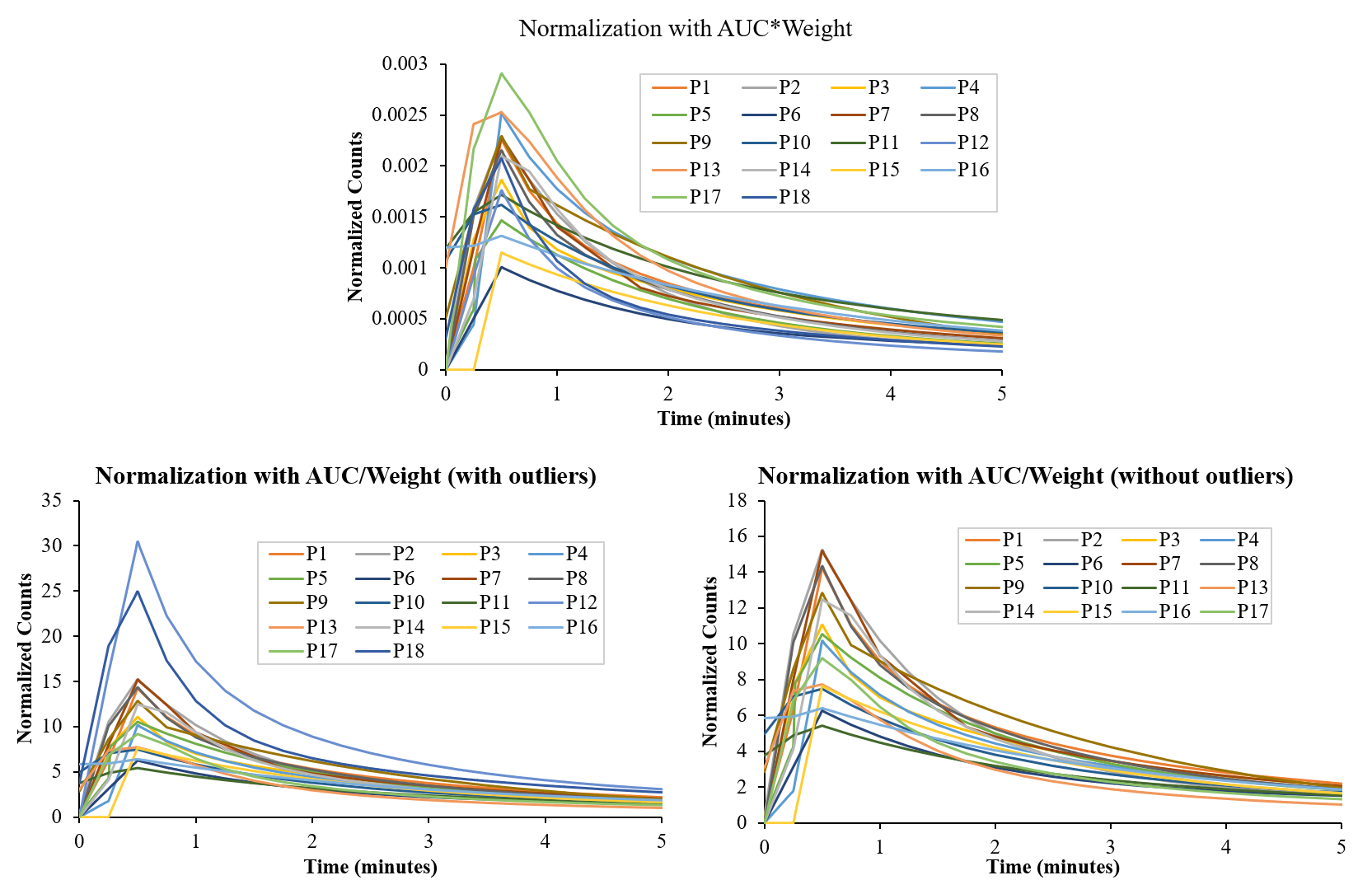


***Figure S6: Comparison of normalization by AUC*** $\boldsymbol{\times}$ ***Weight_subject_ versus AUC / Weight_subject_***

While normalizing with AUC/ Weight_subject_, we noticed some variability due to outliers (shown in the Figure S6). We removed the outliers and generated the PBIF. We then run the kinetic analysis for few patients using the PSAIF and the two PBIFs. From the results shown in Table S2, we noticed a greater % Difference between PSAIF- and PBIF-based V_T_ estimates when using normalization Dose_Injected_ / Weight_subject_ as well as AUC / Weight_subject_

***Table S2: % Difference between patient-specific and population-based kinetic parameters***

| **Brain Regions** | **Normalization Techniques** | | | |
| --- | --- | --- | --- | --- |
|  | **AUC*Weight** | **AUC/Weight** | **Dose*Weight** | **Dose/Weight** |
| White Matter | 10.3 | 13.5 | 10.4 | -19.6 |
| Cerebellum | 11.5 | 15.1 | 10.9 | -19.1 |
| Thalamus | 11.3 | 15.6 | 11.5 | -18.7 |
| Caudate | 13.1 | 25.4 | 20.7 | -12.1 |
| Putamen | 11.4 | 15.8 | 11.6 | -18.7 |
| Pallidum | 11.1 | 15.4 | 11.3 | -18.9 |
| Hippocampus | 11.3 | 15.7 | 11.5 | -18.8 |
| Amygdala | 11.2 | 14.3 | 11.4 | -18.9 |

1. **Kinetic Modelling and Evaluation of V_T_ reproducibility**

The kinetic modelling was done with Logan V_T_ graphical model. The linearity time *t** for the plot was determined using a 10% maximum admissible error criterion as described by Ichise et al (30). (27). An example of the Logan fit for a sample patient using both PSAIF and PBIF is shown in Supplementary Figure S5.

We also evaluated the reproducibility of the V_T_ generated with PBIF to that of PSAIF using the estimated *t**, and other goodness of fit criteria (AIC, R^2^ and % standard error (SE)). The result is shown in Figure S6. There is no difference between the V_T_s generated by PBIF and PSAIF based on these criteria.


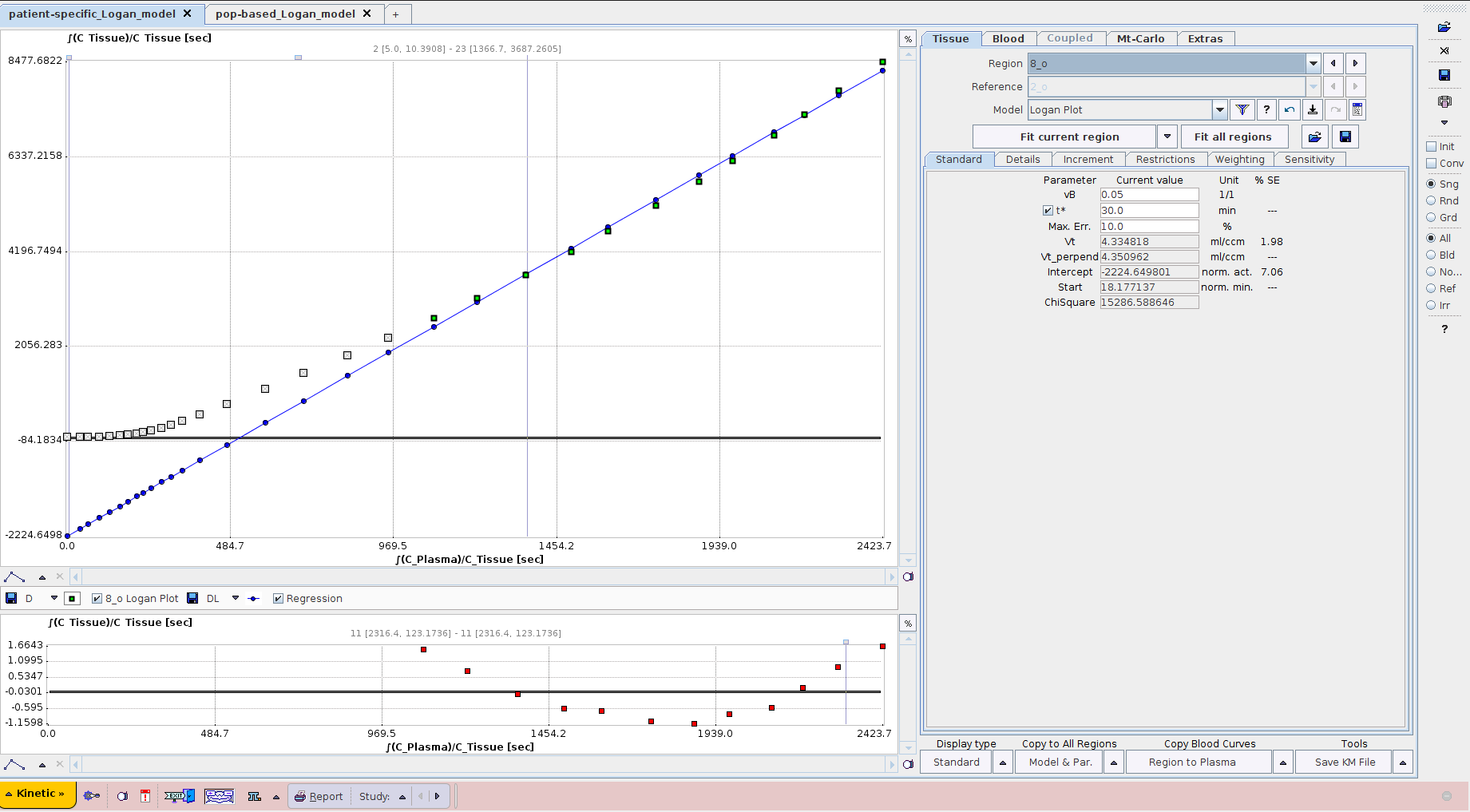


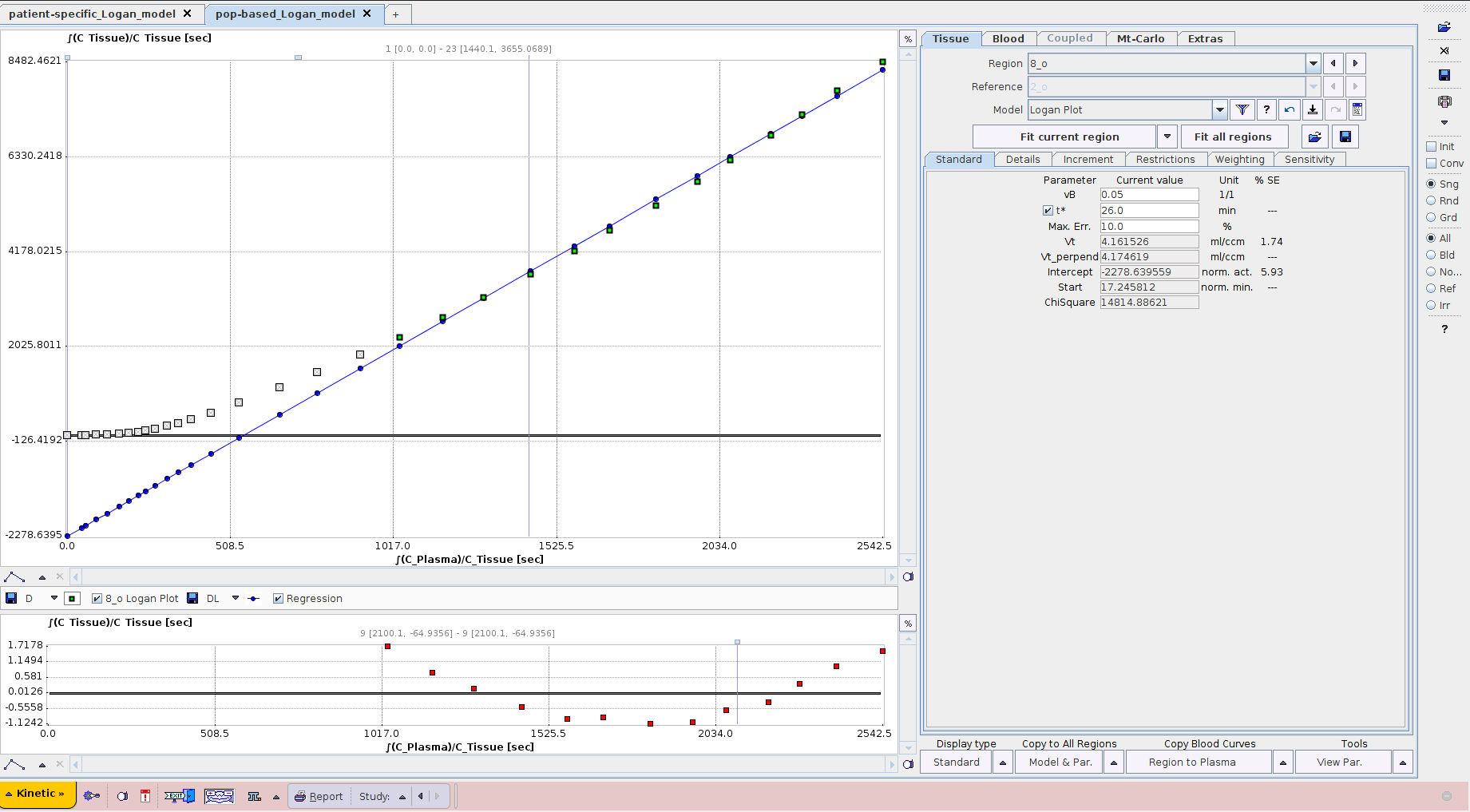


***Figure S7****: Example of the Logan V_T_ plot generated by the patient-specific input function (upper row) and the population-based input function (lower row).*


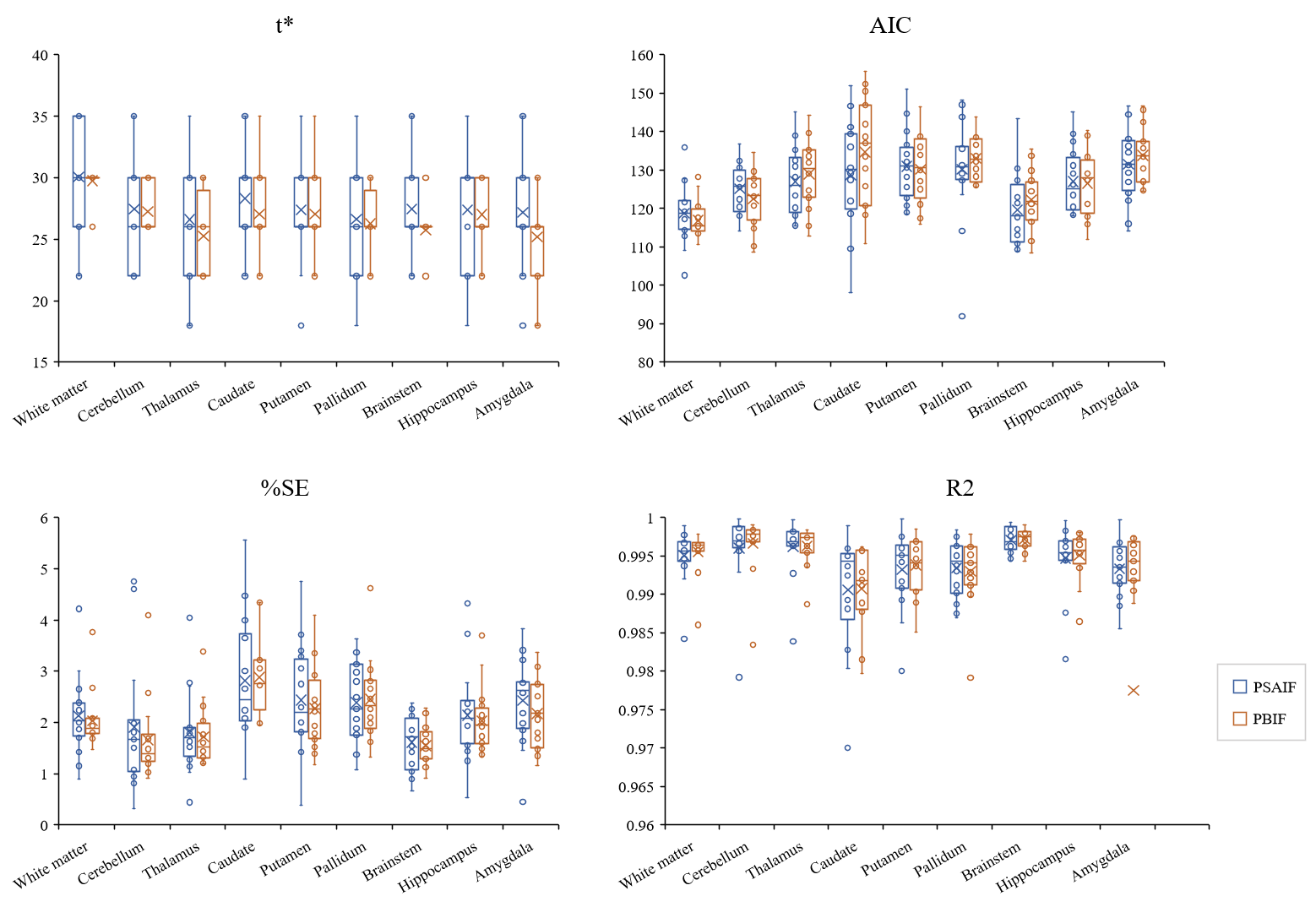


***Figure S8*** *Comparing the PBIF-estimated V_T_ and PSAIF-estimated V_T_ using the goodness of fit criteria.*
